# The art of the deal: Deciphering the endowment effect from traders’ eyes

**DOI:** 10.1126/sciadv.adf2115

**Published:** 2023-08-23

**Authors:** Feng Sheng, Ruining Wang, Zexian Liang, Xiaoyi Wang, Michael L. Platt

**Affiliations:** ^1^ School of Management, Zhejiang University, Hangzhou, ZJ 310058, China.; ^2^ Neuromanagement Laboratory, Zhejiang University, Hangzhou, ZJ 310058, China.; ^3^ State Key Lab of Brain-Machine Intelligence, Zhejiang University, Hangzhou, ZJ 310058, China.; ^4^ MOE Frontier Science Center for Brain Science & Brain-Machine Integration, Zhejiang University, Hangzhou, ZJ 310058, China.; ^5^ Wharton Neuroscience Initiative, Wharton School, University of Pennsylvania, Philadelphia, PA 19104, USA.; ^6^ Department of Neuroscience, Perelman School of Medicine, University of Pennsylvania, Philadelphia, PA 19104, USA.; ^7^ Department of Psychology, University of Pennsylvania, Philadelphia, PA 19104, USA.; ^8^ Marketing Department, Wharton School, University of Pennsylvania, Philadelphia, PA 19104, USA.

## Abstract

People are often reluctant to trade, a reticence attributed to the endowment effect. The prevailing account attributes the endowment effect to valuation-related bias, manifesting as sellers valuing goods more than buyers, whereas an alternative account attributes it to response-related bias, manifesting as both buyers and sellers tending to stick to the status quo. Here, by tracking and modeling eye activity of buyers and sellers during trading, we accommodate both views within an evidence-accumulation framework. We find that valuation-related bias is indexed by asymmetric attentional allocation between buyers and sellers, whereas response-related bias is indexed by arousal-linked pupillary reactivity. A deal emerges when both buyers and sellers attend to their potential gains and dilate their pupils. Our study provides preliminary evidence for our computational framework of the dynamic processes mediating the endowment effect and identifies physiological biomarkers of deal-making.

## INTRODUCTION

From barters to stock markets, from goods to services, from labor to intellectual property, the modern economy depends on trading. Despite the importance of trading, people are often reluctant to part with what they have to trade for what they do not have, a phenomenon termed the endowment effect (*1*–*3*). For example, when people are randomly endowed with either a tranche of cash or a lottery ticket of a comparable value, most refuse the subsequent opportunity to trade what they now have for the alternative. The cash holders will not buy the lottery ticket unless there is a discount, and the ticket holders will not sell it unless there is a premium, leading to a transaction failure (*4*). In the past four decades, the endowment effect has been demonstrated extensively in both laboratory (*1*, *2*) and field studies (*5*, *6*), observed in humans and animals (*7*, *8*), and revealed in behavior and brain activity (*9*–*11*). The endowment effect challenges the efficiency of the free market and defies easy explanation by the basic assumptions of economics.

Despite the significance of the endowment effect, the underlying mechanisms remain controversial. The prevailing view attributes the endowment effect to valuation-related bias. For example, the seminal account attributes the endowment effect to loss aversion (*1*–*3*), a tendency to amplify the subjective value of potential losses over potential gains (*12*). According to this account, sellers value goods more than do buyers, because goods are potential losses for sellers but potential gains for buyers. In addition to loss aversion, many other psychological factors, including a sense of ownership (*13*–*15*) or the desire to make a good deal (*16*), have also been identified as motivations driving sellers to overvalue goods relative to buyers [see (*17*) for a review]. By contrast, an alternative view attributes the endowment effect to a response-related bias between sellers and buyers (*18*–*20*) that is presumably driven by an inertia to maintain the status quo (*19*). According to this view, buyers and sellers are both hesitant to trade because that would alter the status quo, and thus discounts and premiums are required for them to overcome this reluctance. These two views are assumed to be related and are not readily distinguished from choice alone (*21*).

In most cases, decisions are not made instantaneously but instead emerge over time. Recent evidence indicates that economic decisions, just like perceptual decisions (*22*), are made through a process of evidence accumulation over time that culminates in a response (*23*, *24*), which is captured by computational models like the drift diffusion model (DDM) (*25*). If trading emerges from a similar evidence-accumulation process, valuation-related bias and response-related bias should be distinct. Theoretically, valuation-related bias should affect how information is evaluated over time, while response-related bias should affect how much evidence must be accumulated to trigger a decision (*24*). Specifically, in trading, valuation-related bias should manifest as sellers overweighing goods relative to buyers during evidence accumulation, whereas response-related bias should manifest as both sellers and buyers requiring more evidence to accept a deal than to reject it. Thus, by modeling buying and selling decisions as evidence-accumulation processes, it should be possible to accommodate valuation-related bias and response-related bias in a unified computational framework. Recently, a growing corpus of studies has applied an evidence-accumulation framework to value-based decision-making and dissociated valuation-related bias and response-related bias contributing to risky choice (*24*, *26*), intertemporal choice (*27*), consumption choice (*28*, *29*), and altruistic choice (*30*).

Decision-making as an evidence-accumulation process is not merely hypothetical and descriptive but is evident in neurophysiological activity (*31*, *32*) and detectable from gaze allocation and pupil dilation of decision-makers (*24*, *33*). Therefore, by further coupling evidence-accumulation modeling with eye-tracking and pupillometry, it should be possible to identify physiological biomarkers of valuation-related bias and response-related bias that may underlie the endowment effect. By using this approach, we aim to construct a neurobiologically plausible computational framework for understanding trading decisions.

Where people look while they make decisions reveals the temporal dynamics of information sampling that support the otherwise-concealed process of evidence accumulation. Gaze dynamics during decision-making both reflect and shape underlying biases. A growing body of studies demonstrates that people tend to look more at information they value more, and this gaze bias, in turn, amplifies the value of the attended information, overall manifesting as a positive feedback loop between gaze allocation and valuation [see (*34*) for an initial demonstration and (*35*) for a recent review]. Within the evidence-accumulation framework, the dynamic modulation of gaze allocation on valuation is formalized by the attentional DDM (aDDM) (*36*, *37*). By applying this modeling approach to risky decisions involving trade-offs between potential gains and losses, we recently showed that subjective magnification of losses relative to gains, or loss aversion, is indexed by gaze bias toward losses. In the literature on the endowment effect, sellers, relative to buyers, have been found to allocate more gaze fixations to value-increasing attributes of goods, a bias associated with the manifestation of the endowment effect (*38*). Therefore, we hypothesize that overvaluation of goods by sellers relative to buyers will be associated with gaze bias toward goods by sellers relative to buyers.

Changes in pupil size reflect, in part, fluctuations in the norepinephrine-mediated arousal system (*39*). During decision-making, pupil dilation indexes mental effort that is often accompanied by physiological arousal (*40*). Within the evidence-accumulation framework, pupil dilation is associated with the amount of evidence required to trigger a decision (*24*, *33*). Accordingly, choosing against a default response tendency requires more evidence and is often accompanied by greater pupil dilation (*24*, *33*). Thus, we hypothesize that if both buyers and sellers tend to maintain the status quo rather than trading, their pupils will dilate when they overcome this inertia and make a deal.

**Fig. 1.**
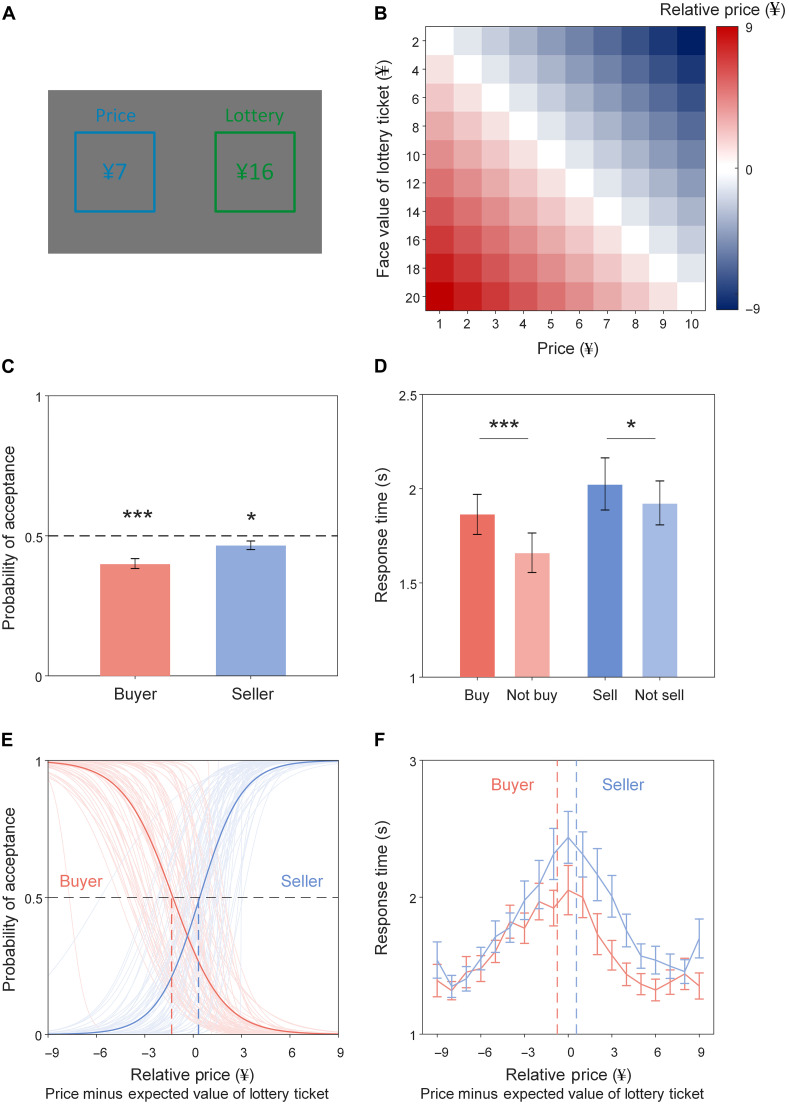
Endowment effect in choices and response times in the trading task. (**A**) The trading task. The example displays an offer to buy a lottery ticket of ¥16 face value at the price of ¥7 in the buyer block or sell it at the same price in a seller block. Participants chose to accept or reject an offer by pressing one of two keys. The words “Price” and “Lottery” were displayed in Chinese to our sample. (**B**) The set of offers presented. Relative price was calculated as the difference between the price and the expected value of a lottery ticket (i.e., half of the face value). Probability of acceptance (**C**) and response times of accepting and rejecting offers (**D**). Error bars denote SEM. *P < 0.05. ***P < 0.001. (**E**) Probability of acceptance conditional on relative price. Light and dark curves were fitted with logistic regressions performed at the individual and group levels, respectively. Red and blue dashed vertical lines indicate mean relative prices eliciting 50% probability of acceptance for buyers and sellers, respectively. (**F**) Response times conditional on relative price. Red and blue dashed vertical lines indicate mean relative prices eliciting the longest response times for buyers and sellers, respectively. Error bars denote SEM.

To test these hypotheses, we studied decisions to buy and sell lottery tickets in a laboratory experiment. Each lottery ticket had a visible face value, while the actual amount of money for which it could be redeemed was a number randomly selected between zero and its face value, invisible to either buyers or sellers. Thus, the expected value of each lottery ticket was half its face value. In the experiment, participants (*N* = 64) played the roles of buyer and seller in two separate blocks, while their gaze and pupil diameter were monitored using a video-based eye-tracking system. In each block, participants were presented with many offers on the computer screen that allowed them to buy or sell lottery tickets with varying face values at specific prices (Fig. 1A). For each offer, participants decided whether to buy or not in the buyer block and whether to sell or not in the seller block by pressing one of two keys. The face values of lottery tickets ranged from ¥2 to ¥20, in ¥2 increments, and prices ranged from ¥1 to ¥10, in ¥1 increment, resulting in 100 unique offers within each block (Fig. 1B, see Materials and Methods for details). We applied the DDM to quantitatively decompose the decision process and identify physiological biomarkers of any potential valuation-related bias or response-related bias between buyers and sellers, with the goal of unveiling mechanisms underlying the endowment effect in trading and its resolution to make a deal.

## RESULTS

### The endowment effect manifests in both choices and response times

We observed substantial endowment effects in our participants. Overall, buyers chose to buy less than 50% of all lottery tickets (Fig. 1C, mean ± SD = 40.20 ± 14.04%; *t*_63_ = −5.582, *P* < 0.001). Accordingly, when acting as sellers, these same participants should choose to sell more than half of all lottery tickets for their preferences to remain consistent across roles. We found that sellers chose to sell less than 50% of all lottery tickets (Fig. 1C, mean ± SD = 46.72 ± 12.24%; *t*_63_ = −2.144, *P* = 0.036), revealing an inconsistency in their preferences. Sellers made overall slower decisions (Fig. 1D, mean ± SD = 1.955 ± 0.986) than did buyers (mean ± SD = 1.712 ± 0.826; *t*_63_ = 3.775, *P* < 0.001). Notably, buyers were slower to choose to buy than to choose not to buy (Fig. 1D, Δmean ± SD = 0.203 ± 0.426; *t*_63_ = 3.818, *P* < 0.001), suggesting hesitancy to acquire lottery tickets at most prices, but sellers were slower to choose to sell than to choose not to sell (Fig. 1D, Δmean ± SD = 0.101 ± 0.384; *t*_63_ = 2.095, *P* = 0.040), indicating a reluctance to part with lottery tickets for the same set of prices. Thus, we observed an incongruity in both choices and response times when participants acted as buyers and sellers.

To better understand these inconsistencies, we analyzed how choices and response times varied as a function of price while lottery ticket face value was controlled (fig. S1). Specifically, we calculated the relative price for each offer by subtracting the expected value of the lottery ticket (i.e., half of the face value) from the price (Fig. 1B). We found that as relative prices rose, the probability of buying systematically decreased (Fig. 1E, red curve; mixed-level logistic regression, *B* = −0.893, SE = 0.041, *t*_6398_ = −22.029, *P* < 0.001) and the probability of selling increased (blue curve; *B* = 1.067, SE = 0.051, *t*_6398_ = 20.853, *P* < 0.001; see table S1 for more details). The relative price that elicited a 50% probability of accepting an offer—the indifference point—was higher for sellers than for buyers (Fig. 1E, blue and red dashed lines; Δmean ± SD = 1.665 ± 1.783, *t*_63_ = 7.470, *P* < 0.001). Specifically, buyers required a surplus significantly larger than zero to accept an offer (Fig. 1E, zero minus indifference point; mean ± SD = 1.338 ± 1.891; *t*_63_ = 5.660, *P* < 0.001), but sellers did not require a surplus significantly larger than zero to accept an offer (indifference point minus zero, mean ± SD = 0.327 ± 1.572; *t*_63_ = 1.664, *P* = 0.101). The surplus required by sellers was significantly smaller than the surplus required by buyers (*t*_63_ = −2.709, *P* = 0.009). Similarly, we found that the relative price that elicited the longest response times—a chronometric estimate of the indifference point (*41*)—was marginally, but not significantly, larger for sellers (Fig. 1F, blue dashed line, mean ± SD = 0.563 ± 4.342) than for buyers (red dashed lines, mean ± SD = −0.750 ± 3.928; *t*_63_ = 1.922, *P* = 0.059), although neither price was significantly different from zero (both *P* values > 0.131). Together, our data revealed that the price sellers were willing to accept parting with a lottery ticket higher than the price buyers were willing to pay to acquire the lottery ticket, consistent with typical price gaps in the endowment effect revealed in prior studies (*4*).

### Valuation-related bias and response-related bias are dissociable during trading

**Fig. 2.**
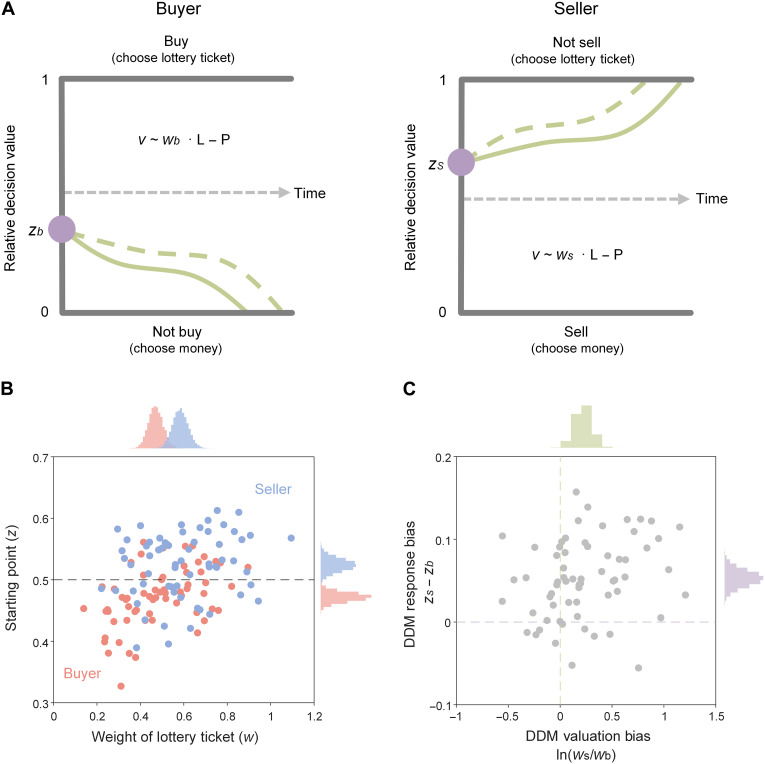
DDM unveils valuation-related bias and response-related bias during trading decisions. (**A**) Illustration of the DDM applied to the trading task. Purple dots indicate starting points for evidence accumulation. Green curves indicate schematic evidence-accumulation processes (with smoothed noise). An increase in the weight of lottery ticket would lead the evidence-accumulation processes to shift from the solid green curves to the dashed green curves. (**B**) Estimated starting points and lottery weights of buyers (red) and sellers (blue), respectively. (**C**) Calculated DDM valuation bias and DDM response bias. Each dot represents a participant. Histograms above the upper border and to the right border indicate distributions of the group estimates.

We next developed and applied a joint DDM to account for the decisions made by both buyers and sellers. When an offer like the one in Fig. 1A was presented, buyers and sellers were faced with similar options. Buyers were presented with the option to keep the amount of money depicted by the price or to trade it for a lottery ticket of the depicted face value. Similarly, sellers were presented with the option to keep the lottery ticket or to trade it for the amount of money indicated by the price. Thus, choosing between the amount of money and the lottery ticket was the same for buyers and sellers, but given the status quo, choosing to keep the money was the default for buyers, whereas choosing to keep the lottery ticket was the default for sellers. Within this framework, we assumed that each trading decision emerged from a noisy evidence-accumulation process that drifted between two response boundaries (Fig. 2A). When the upper boundary (denoted by 1) was reached, the accumulation process would end with choosing the lottery ticket. That is, buyers would choose to buy, and sellers would choose not to sell. By contrast, when the bottom boundary (denoted by 0) was reached, the accumulation process would end with choosing the money. That is, buyers would choose not to buy, and sellers would choose to sell.

The speed of evidence accumulation, or drift rate (*v*), was determined by the linear combination of the price (*P*) and the expected value of the lottery ticket (denoted by *L*, i.e., half of its face value) multiplied by a weighting coefficient *w*. Critically, the weighting coefficient for the lottery ticket was assumed to be independent when a participant was a buyer (*w*_b_) and a seller (*w*_s_). We calculated the logarithmic ratio of the weights assigned to the lottery ticket by seller versus buyer, ln (*w*_s_/*w*_b_), to index valuation-related bias, or what we termed DDM valuation bias. Response-related bias, or DDM response bias, by contrast, is reflected in the starting point of the evidence-accumulation process. A starting point halfway between the two response boundaries indicates no a priori bias toward either option. The tendency to maintain the status quo would shift the starting point of the evidence-accumulation process for a buyer (*z*_b_) toward the lower boundary of not buying and shift the evidence-accumulation process for a seller (*z*_s_) toward the upper boundary of not selling. Consequently, the difference between the starting points as seller and buyer, *z*_s_ − *z*_b_, indexes DDM response bias for each participant. In addition, we included an intercept *b* in the drift rate to account for variance in choices and response times that was irrelevant to price and expected value of lottery ticket, and a parameter *a*, or boundary separation, to scale the distance between the upper and the lower boundaries relative to noises, both of which (i.e., *b* and *a*) were assumed to be independent for buyer and seller. See Materials and Methods for the complete model.

In this model, both lottery weights and starting points are reflected not only in choice but also in response time. A larger weight for a lottery ticket would lead to a higher probability and shorter time to choose not to sell and a lower probability and longer time to choose not to buy, illustrated by the shift of accumulation processes from the solid lines to the dashed lines in Fig. 2A. A higher starting point, or a starting point closer to the upper response boundary, would have a similar effect, illustrated by the contrast of the starting points between seller and buyer in Fig. 2A. By fitting this model simultaneously to observed choices and response times using a hierarchical Bayesian approach (see Materials and Methods for details), we obtained estimates for the starting points and the weighting coefficients of the lottery ticket when each participant acted as either buyer or seller (Fig. 2B and table S2, DDM1). The fitted model well predicted the actual choices and response times. Across individuals, the choices predicted by the model (fig. S2A) were consistent with 92.60 ± 2.41% (mean ± SD%) of actual choices, and the response times predicted by the model (fig. S2B) were modestly correlated with actual response times [mean Pearson’s correlation coefficient (*r*) = 0.347, SD = 0.131; *t*_63_ = 21.206, *P* < 0.001]. On the basis of these parameter estimates, we then calculated DDM valuation bias and DDM response bias for each participant (Fig. 2C) and the overall population (table S2, green and purple columns).

At the population level, we observed a DDM valuation bias significantly larger than zero (Fig. 2C, green histogram, mean = 0.216, 95% credible interval: 0.059 to 0.376), consistent with sellers assigning a larger weight to lottery ticket than did buyers. We also found a DDM response bias significantly larger than zero (Fig. 2C, purple histogram, mean = 0.052, 95% credible interval: 0.033 to 0.072), consistent with higher evidence-accumulation starting points for sellers than for buyers. Specifically, starting points were significantly lower than the midpoint (0.5) in buyers (Fig. 2B, red dots, mean = 0.471, 95% credible interval: 0.456 to 0.486) but higher than the midpoint in sellers (blue dots, mean = 0.523, 95% credible interval: 0.502 to 0.545), indicating a tendency to maintain the status quo in both buyers and sellers.

Across individuals, we observed substantial heterogeneity in DDM valuation bias, ranging from −0.558 to 1.207, and DDM response bias, ranging from −0.055 to 0.157. Moreover, these two biases were marginally, but not significantly, correlated with each other (Fig. 2C, Pearson’s *r* = 0.239, *P* = 0.058). To clarify the independence of the two biases, we compared this model (DDM1), which included both DDM valuation bias and DDM response bias, with two reduced models, namely, one without DDM valuation bias (DDM2, set *w*_s_ = *w*_b_) and another without DDM response bias (DDM3, set *z*_s_ = *z*_b_). We found that the full model outperformed the other two reduced models [both ΔDICs (deviance information criteria) <−146; see table S2 for more details]. Thus, although DDM valuation bias and DDM response bias were weakly correlated across individuals, they were both necessary to account for the observed variation in choices and response times. See more results in the “Intercept” and “Boundary separation” sections in Supplementary Text.

### Valuation-related bias manifests in gaze allocation during trading decisions

**Fig. 3.**
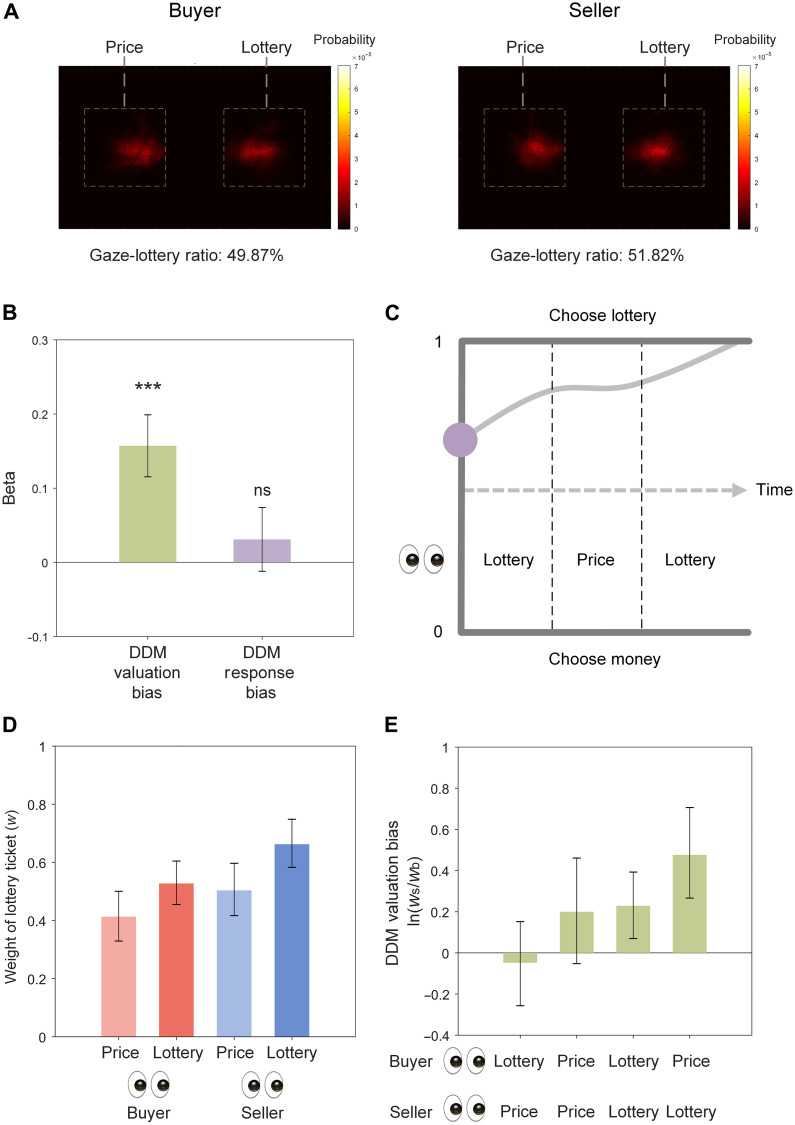
Buyers and sellers selectively attend to prices and lottery tickets. (**A**) Probability of gaze allocation to price and lottery ticket when participants acted as buyers (left) or sellers (right), respectively. (**B**) Gaze-lottery ratio predicted by DDM valuation bias and DDM response bias across participants using regression. ***P < 0.001. ns, nonsignificant. (**C**) Illustration of the aDDM applied to the trading task. The drift rate of evidence accumulation (with smoothed noise) varies with gaze allocated to the price versus the lottery ticket. (**D**) Estimates of the weight assigned to the lottery ticket by buyers (red) and sellers (blue) when either the price (light) or the lottery ticket (dark) was fixated. (**E**) DDM valuation bias calculated by coupling gaze data for both the buyer role and the seller role played by each participant.

We next examined participants’ gaze allocation during trading. On average, before a decision was made, participants made 3.359 ± 1.156 (mean ± SD) fixations on the displayed price and lottery ticket when acting as sellers (fig. S3), more than the number of fixations they made when acting as buyers (mean ± SD = 3.087 ± 0.968; *t*_63_ = 3.152, *P* = 0.002), which was consistent with the longer time sellers took to make decisions. To explore potential gaze bias between the price and the lottery ticket, for each trial, we calculated gaze-lottery ratio as the time spent fixating the lottery ticket relative to the total time spent fixating either the price or the lottery ticket and computed the average when each participant acted as buyer or seller, respectively. We found that gaze-lottery ratio was slightly, but not significantly, larger in sellers (Fig. 3A, mean ± SD = 51.82 ± 11.48%) than in buyers (mean ± SD = 49.87 ± 11.99%; *t*_63_ = 1.879, *P* = 0.065).

To examine the relationship of gaze allocation to the endowment effect, we calculated total gaze bias from the difference of gaze-lottery ratio between seller and buyer for each participant and regressed it simultaneously on DDM valuation bias and DDM response bias across participants. We found that individual differences in total gaze bias were correlated with individual differences in DDM valuation bias (Fig. 3B, *B* = 0.157, SE = 0.042, *t*_61_ = 3.760, *P* < 0.001) but not individual differences in DDM response bias (*B* = 0.031, SE = 0.043, *t*_61_ = 0.721, *P* = 0.474; see the yellow column of table S3). The predictive power of DDM valuation bias on total gaze bias was 5.072 times larger than that of DDM response bias. These findings link total gaze bias to valuation-related bias, as in our prior study on loss aversion (*24*).

We next asked whether the relationship between total gaze bias and valuation-related bias could be elaborated in an aDDM framework. That is, we hypothesized that the weight assigned to a lottery ticket during evidence accumulation would be amplified when gaze was directed at it and attenuated when gaze was directed at the price. To test this hypothesis, we extended our current DDM to a variant of aDDM (aDDM1) that allowed the weight of the lottery ticket to vary with gaze allocation on the price versus the lottery ticket (Fig. 3C, see Materials and Methods for details). We found that this model fit choices and response times better than our DDM (DDM1) that did not incorporate gaze information (ΔDIC = −47, table S4). As hypothesized, the weight buyers assigned to a lottery ticket when they gazed at the price was just 0.787 of the weight when they gazed at the lottery ticket (Fig. 3D, light versus dark red bar; 95% credible interval: 0.608 to 0.988), and the weight sellers assigned to a lottery ticket when they gazed at the price was just 0.762 of the weight when they gazed at the lottery ticket (Fig. 3D, light versus dark blue bar, 95% credible interval: 0.617 to 0.920).

Consequently, coupling gaze data for both the buyer role and the seller role played by each participant for each offer, we hypothesized that we would observe four different sizes of DDM valuation bias contingent on where the coupled buyer and seller look (Fig. 3E). DDM valuation bias would be the largest when the seller and the buyer both attended to their potential losses—that is, when the seller attended to the lottery ticket and the buyer attended to the price (Fig. 3E, the fourth bar, mean = 0.476, 95% credible interval: 0.266 to 0.706). By contrast, DDM valuation bias would be smallest when the buyer and the seller both attended to their potential gains—that is, when the buyer attended to the lottery ticket and the seller attended to the price (mean = −0.049, 95% credible interval = −0.257 to 0.152). The DDM valuation bias was no larger than zero under this condition (i.e., the 95% credible interval included zero). In the remaining two conditions, when both the buyer and the seller attended to the price, DDM valuation bias was not significantly larger than zero (Fig. 3E, the second bar, mean = 0.200, 95% credible interval = −0.052 to 0.461), although the lower bound of the 95% credible interval was close to zero. When both the buyer and the seller attended to the lottery ticket, DDM valuation bias was significantly larger than zero (Fig. 3E, the third bar, mean = 0.228, 95% credible interval = 0.070 to 0.393). See table S4 for more details. These analyses suggest that valuation-related bias partially but not fully depended on gaze bias. Even when both buyer and seller attended to the same information (i.e., lottery ticket), they could value the goods differently.

In addition to where participants looked more, we also examined where the participants looked first. For each participant, we calculated first-gaze-lottery probability as the probability of the first fixation on lottery tickets. We found that first-gaze-lottery probability was lower than 0.5 in both buyers (fig. S4A, mean ± SD = 0.198 ± 0.192; *t*_63_ = −12.603, *P* < 0.001) and sellers (mean ± SD = 0.346 ± 0.246; *t*_63_ = −5.016, *P* < 0.001), suggesting that both roles tended to look first at prices rather than lottery tickets. Notably, relative to buyers, sellers were less likely to look first at prices—In other words, sellers were more likely to look first at lottery tickets (fig. S4A, *t*_63_ = 5.807, *P* < 0.001), revealing a disparity between sellers and buyers in first fixation. We calculated first-gaze bias from the difference of first-gaze-lottery probability between seller and buyer for each participant. We found that first-gaze bias was significantly correlated with total gaze bias across individuals (Pearson’s *r* = 0.335, *P* = 0.007), suggesting that those tending to look first at lottery tickets when acting as sellers relative to when acting as buyers also tended to look more at lottery tickets than prices overall in the seller block than in the buyer block.

To examine the relationship of first-gaze bias to the endowment effect, we regressed it simultaneously on DDM valuation bias and DDM response bias across participants. Similar to total gaze bias, we found that individual differences in first-gaze bias were correlated with individual differences in DDM valuation bias (fig. S4B, *B* = 0.240, SE = 0.110, *t*_61_ = 2.184, *P* = 0.033) but not individual differences in DDM response bias (*B* = 0.105, SE = 0.113, *t*_61_ = 0.931, *P* = 0.355; see the yellow column of table S5). Thus, DDM valuation bias manifested in both first-gaze bias and total gaze bias. To further clarify the relationships among the three variables, we regressed DDM valuation bias simultaneously on the two gaze biases. We found that total gaze bias remained a significant predictor of DDM valuation bias (*B* = 0.937, SE = 0.276, *t*_61_ = 3.392, *P* = 0.001), but first-gaze bias was not (*B* = 0.325, SE = 0.234, *t*_61_ = 1.390, *P* = 0.170; see the yellow column of table S6). These findings suggest that the association between valuation-related bias and first-gaze bias is mediated by total gaze bias. See the “First gaze” section in Supplementary Text for modeling analyses on first gaze.

### Response-related bias manifests in pupil dilation when choosing to trade

**Fig. 4.**
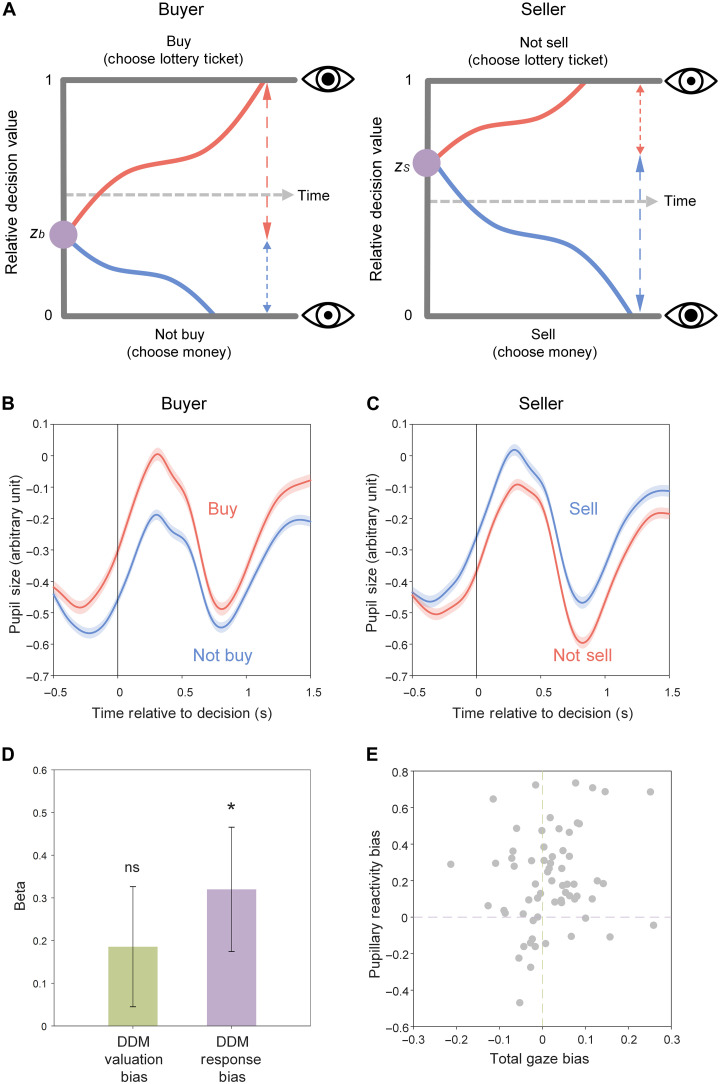
Pupil dynamics reveal response-related bias in buyers and sellers. (**A**) Illustration of hypothesized relationships between choices and pupil sizes within the DDM framework. (**B** and **C**) Pupil size as a function of time anchored on the decision (*t* = 0) to accept or reject offers for buyers and sellers. Shaded areas indicate SE. (**D**) Pupillary reactivity bias predicted by DDM valuation bias and DDM response bias across participants. *P < 0.05; ns, nonsignificant. (**E**) Scatter plot of total gaze bias and pupil reactivity bias. Each dot indicates a participant.

We next examined changes in pupil size during the trading process. We hypothesized that choosing to trade, which would disrupt the status quo, would require more mental effort and consequently evoke greater pupil dilation. Formally, our DDM framework assumed that larger pupil dilation reflects a larger amount of evidence required to trigger the choice of accepting an offer, which is depicted as a longer journey of the evidence-accumulation process from the starting point to the acceptance boundary (Fig. 4A).

As expected, we observed that pupil size, as a sluggish physiological signal, for accepting versus rejecting an offer, began to diverge about 0.5 s before a decision was made, and continued to diverge for at least 1.5 s after decisions, in both buyers and sellers (Fig. 4, B and C, and fig. S5). Following prior studies (*24*, *33*), we calculated decision-related pupil size from mean pupil size in the time window from −0.5 s before to 1.5 s after decision on each trial and then averaged across trials for each participant separately playing as buyer and seller. Overall, participants showed larger decision-related pupil size when they chose to buy than when they chose not to buy (Fig. 4B, Δmean ± SD = 0.146 ± 0.231, *t*_63_ = 5.075, *P* < 0.001) and also when they chose to sell than when they chose not to sell (Fig. 4C, Δmean ± SD = 0.056 ± 0.193, *t*_63_ = 2.316, *P* = 0.024). In other words, when acting as buyers, participants showed larger pupil dilation for choosing lottery tickets than choosing money, but when acting as sellers, participants showed larger pupil dilation for choosing money than choosing lottery tickets. These results suggest that accepting and rejecting an offer are asymmetric decisions for both buyers and sellers, and choosing lottery tickets and choosing money are diametric decisions for buyers versus sellers.

Notably, the offers accepted by buyers, relative to the offers rejected by them, were usually of lower prices and higher expected values of lottery tickets, while the offers accepted by sellers, relative to the offers rejected by them, were usually of higher prices and lower expected values of lottery tickets (fig. S1A). Thus, the observed differences in decision-related pupil size between accepted and rejected offers could simply reflect differences in price or expected value of lottery ticket rather than the choice itself. To test this possibility, we performed a mixed-level regression, for buyers and sellers, respectively, to predict decision-related pupil size with choice (accept, 1; reject, 0), price, and expected value of lottery ticket. We found that for buyers, choice significantly predicted decision-related pupil size (table S7; light red column, *B* = 0.171, SE = 0.033, *t*_6278_ = 5.232, *P* < 0.001), while price was just marginally predictive (*B* = 0.089, SE = 0.051, *t*_6278_ = 1.744, *P* = 0.081), and expected value of lottery ticket was not predictive at all (*B* = −0.003, SE = 0.034, *t*_6278_ = −0.077, *P* = 0.939). For sellers, choice predicted decision-related pupil size (table S7; light blue column, *B* = 0.080, SE = 0.036, *t*_6288_ = 2.210, *P* = 0.027), but neither price (*B* = −0.031, SE = 0.052, *t*_6288_ = −0.587, *P* = 0.557) nor expected value of lottery ticket (*B* = 0.036, SE = 0.034, *t*_6288_ = 1.030, *P* = 0.303) did. Thus, decision-related pupil size is primarily associated with the choice itself rather than the price or the expected value of the lottery ticket in an offer.

In our study, the asymmetry between accepting and rejecting offers was not just observed in pupillary reactivity but also in response time, with both sellers and buyers being slower to accept than reject offers (Fig. 1D). Within our computational framework (Fig. 4A), response time is hypothesized to be correlated with decision-related pupil size because both are associated with the amount of evidence accumulated to trigger a choice. As hypothesized, we found that decision-related pupil size was predicted by response time in both buyers (table S7; mixed-level regression, *B* = 6.440, SE = 0.920, *t*_6280_ = 7.001, *P* < 0.001) and sellers (*B* = 3.831, SE = 0.716, *t*_6290_ = 5.354, *P* < 0.001). This observation invites the possibility that the differences in decision-related pupil sizes between accepted and rejected offers might simply be attributable to the differences in response times to accept versus reject offers. To test this possibility, we included response time in addition to choice, price, and expected value of lottery ticket, to predict decision-related pupil size using mixed-level regressions, for buyers and sellers, respectively. We found that choice remained a significant predictor of decision-related pupil size for both buyers (table S7; red column, *B* = 0.137, SE = 0.033, *t*_6277_ = 4.175, *P* < 0.001) and sellers (blue column, *B* = 0.079, SE = 0.035, *t*_6287_ = 2.288, *P* = 0.022) when response time was controlled (buyer: *B* = 5.938, SE = 0.855, *t*_6277_ = 6.942, *P* < 0.001; seller, *B* = 3.746, SE = 0.710, *t*_6287_ = 5.277, *P* < 0.001). Notably, response time showed larger predictive power than choice for both buyers (linear contrast of betas, *F*_1,6277_ = 46.010, *P* < 0.001) and sellers (linear contrast of betas, *F*_1.6287_ = 26.262, *P* < 0.001). Together, these findings indicate that response time explains the largest proportion of variance in decision-related pupil size but does not fully account for the variance explained by choice itself.

We then tested whether decision-related pupil size reflected DDM valuation bias or DDM response bias. We calculated pupillary reactivity bias from the sum of differential decision-related pupil sizes to accept versus reject offers across buyer and seller roles for each participant and then regressed it on DDM valuation bias and DDM response bias. We found that individual differences in pupillary reactivity bias were predicted by their DDM response biases (Fig. 4D, *B* = 0.319, SE = 0.144, *t*_61_ = 2.217, *P* = 0.030) but not their DDM valuation biases (*B* = 0.190, SE = 0.140, *t*_61_ = 1.364, *P* = 0.177; see the yellow column of table S8 for details). The predictive power of DDM response bias for pupillary reactivity bias was 1.671 times larger than the predictive power of DDM valuation bias. These findings are consistent with our hypothesis that disrupting the status quo to make a trade is accompanied by pupil-linked arousal.

Last, we examined the relationship between pupillary reactivity bias and the two gaze biases in the population. We found that pupillary reactivity bias was correlated with neither total gaze bias (Fig. 4E, Pearson’s *r* = 0.173, *P* = 0.172) nor first-gaze bias (fig. S6, Pearson’s *r* = 0.178, *P* = 0.160). These findings endorse the independence of valuation-related bias and response-related bias as the physiological responses associated with them are distinct.

### Response-related bias cannot be identified by merely modeling choices

By simultaneously modeling choices and response times using a DDM framework, we dissociated valuation-related bias and response-related bias underpinning the endowment effect. A remaining question was whether we could distinguish these biases without modeling response time. It has been formally illustrated that a DDM collapses into a logit model (LM) when response time is not considered (*42*). We therefore developed a variant of LM as a “time-flat” version of our DDM that accommodated both valuation-related and response-related biases [see (*43*) for a related LM built for risky choice]. Specifically, in the model (LM1), we assumed that the subjective value of an offer is determined by the difference between the price (*P*) and the expected value of the lottery ticket (*L*) weighted by a parameter, λ. Similar to our DDM, the logarithmic ratio of the weighting parameters between seller (λ_s_) and buyer (λ_b_), ln(λ_s_/λ_b_), indexes a valuation-related bias, which we termed LM valuation bias. The subjective value was assumed to be translated into the probability of choosing the lottery ticket via a softmax function. Critically, analogous to the starting point in DDM, we introduced a predisposition parameter δ to moderate the range of the softmax function’s output or the possible probability of choosing the lottery ticket. The range is set to be δ to 1 when δ is larger than zero, which indexes a predisposition toward choosing the lottery ticket, and is set to be 0 to 1 + δ when δ is smaller than zero, which indexes a predisposition toward choosing money. Accordingly, the difference between the predisposition parameters of seller (δ_s_) and buyer (δ_b_), δ_s_ − δ_b_, indexes a response-related bias between seller and buyer, which we termed LM response bias. See Materials and Methods for the complete model.

By fitting this model to observed choices, we obtained estimates of the weighting parameters and the predisposition parameters for each participant (fig. S7A; see table S9 for details). The choices predicted by this model were consistent with 89.59 ± 5.19% (mean ± SD) of actual choices (fig. S8), lower than the predictions by the full DDM (fig. S2A; Δmean ± SD = −3.02 ± 5.19%, *t*_63_ = −4.648, *P* < 0.001). This difference suggests that our DDM exploited unique information contained in response times for predicting choices.

On the basis of estimated LM parameters, we then calculated LM valuation bias and LM response bias (fig. S7B) for each participant (see table S9 for details). We observed an LM valuation bias significantly larger than zero (mean ± SD = 0.311 ± 0.704, *t*_63_ = 3.534, *P* < 0.001), suggesting that sellers valued lottery tickets more than buyers did. However, we did not observe an LM response bias significantly different from zero (mean ± SD = 0.008 ± 0.044, *t*_63_ = 1.482, *P* = 0.143). No significant predisposition was observed in either buyers (mean ± SD = −0.002 ± 0.037, *t*_63_ = −0.552, *P* = 0.603) or sellers (mean ± SD = 0.006 ± 0.030, *t*_63_ = 1.494, *P* = 0.140). Consistent with these results, when we performed model comparison, we found that the full model (LM1), which included both LM valuation bias and LM response bias, underperformed the model that constrained LM response bias [LM3, set δ_s_ = δ_b_; ΔBIC (Bayesian information criterion) = 130], although it outperformed the model that constrained LM valuation bias (LM2, set λ_s_ = λ_b_; ΔBIC = −114). That is, it was not necessary to allow the existence of LM response bias to account for the variance in choices. Consequently, we would not identify response-related bias if we solely modeled choices. See the “LM” section in Supplementary Text for more analyses.

## DISCUSSION

The endowment effect has been a puzzle since it was first identified more than four decades ago, with a variety of explanations proposed from the perspectives of economics (*44*), psychology (*17*), and neuroscience (*45*). Here, we provide a cognitive computational framework for the endowment effect and identify distinct physiological mechanisms underlying the decision process during trading. Specifically, by unfolding trading as an evidence-accumulation process, we dissociated the bias embodied in valuation of goods from the bias embodied in response selection between sellers and buyers. Further, we found that valuation-related bias manifests in selective attention of sellers to goods and buyers to prices. Response-related bias is evident in greater pupil dilation when sellers agree to sell and buyers agree to buy. Consequently, synchronized pupil dilation forecasts a successful deal.

Valuation-related bias between sellers and buyers, presumably driven by loss aversion (*1*–*3*) or psychological ownership (*13*–*15*), has been the dominant explanation for the endowment effect since its discovery (*1*–*3*, *13*–*15*). Response-related bias, presumably driven by a tendency to maintain the status quo, by contrast, is a less-prevalent explanation for the endowment effect (*18*–*20*) and is sometimes regarded as a side effect of valuation-related bias (*21*). Here, by simultaneously modeling choices and response times while measuring gaze position and pupil size within a neurobiologically and computationally grounded evidence-accumulation framework, we have identified distinct biases contributing to the endowment effect. That is, valuation-related bias manifests as differential weighting of evidence, whereas response-related bias manifests as disparate amounts of evidence required to trigger decisions. By modeling both choices and response times, our study disentangled the contributions of valuation-related bias and response-related bias to the endowment effect in a way that has not been articulated before. Notably, response-related bias was not identifiable when modeling choices without taking response times into account. Thus, response time contains unique information about the decision process unfolding during trading that is not reflected in choices. Our study thus highlights the significance of response time for understanding the decision process and adds to the growing body of studies using chronometric computational modeling to uncover hidden biases in decision-making (*24*, *26*, *28*–*30*, *46*).

Notably, our decomposition of endowment effect into valuation-related bias and response-related bias identified four types of traders, as illustrated in the four quadrants of Fig. 2C. Most participants (60.94%) showed both a DDM valuation bias and a DDM response bias (quadrant 1). A nonnegligible proportion of participants (23.44%) showed a DDM response bias but a reverse DDM valuation bias (quadrant 2). That is, they were resistant to trading even though they valued goods more when acting as buyers than when acting as sellers. Conversely, a small proportion of participants (9.38%) showed a DDM valuation bias but a reverse DDM response bias (quadrant 4). That is, they were inclined to trade even though they valued goods more when acting as sellers than when acting as buyers. Last, a similarly small proportion of participants (6.25%) showed both DDM valuation bias and DDM response bias but in opposite directions (quadrant 3). That is, they showed no endowment effect. Thus, our framework revealed nuanced heterogeneity of the endowment effect that has previously not been described. See the “LM” section in Supplementary Text for classification of participants based on LM valuation bias and LM response bias.

Different valuation of goods between buyers and sellers has been attributed to distinct styles of information processing in earlier research (*38*, *47*–*49*). Here, using gaze allocation as an index of attentional deployment, we formalized the moment-to-moment impact of visual information processing on evidence accumulation to provide a mechanistic and computational account for the temporal dynamics of valuation-related bias underpinning the endowment effect. Buyers and sellers would value goods most differently when both attend to their potential losses (i.e., price versus goods) and value goods most similarly when both attend to their potential gains (i.e., goods versus price). Notably, when buyers and sellers jointly attend to the goods (Fig. 3E, the third bar), we could still observe larger valuation of goods in sellers than in buyers. These findings suggest that differential valuation of goods between sellers and buyers is partly, but not fully, attributable to attentional allocation, consistent with the theory of a positive feedback loop between valuation and gaze allocation (*34*). That is, most sellers initially value goods more than buyers do either due to loss aversion or for other reasons, which motivates them to preferentially attend to goods. This attention bias, in turn, further amplifies the value of goods for sellers relative to buyers, which manifests as an association between valuation-related bias and gaze bias.

In our study, relative to buyers, sellers were more likely to first look at goods and spend more time looking at goods, both of which were associated with valuation-related bias but not response-related bias. Moreover, we found that the total gaze bias mediated the relationship between first-gaze bias and valuation-related bias. Notably, a recent meta-analysis on attention and decision-making (*50*) found that the choice between two options was affected by the total exposure time of one option relative to the other but not by first-gaze allocation between the two options. By contrast, a recent eye-tracking study applying DDM to value-based decision-making found that the impact of early gaze bias on choice, when not being modeled, could be erroneously attributed to starting point bias (*51*). In our study, however, we found that first gaze accounted for little variance in choices and response times explained by starting point when we modeled the potential modulatory effect of first gaze on starting point (see the “First gaze” section in Supplementary Text). Together, our findings suggest that where one initially looks shapes further visual inspection, thereby amplifying the value of the most often gaze at information—a finding consistent with the seminal work revealing a gaze cascade that begins with biased first gaze (*34*). Nevertheless, in our study, both buyers and sellers tended to look at prices before looking at lottery tickets. We reason that evaluating a lottery ticket involves calculating the trade-off between the magnitude and the probability of potential return, which can be more effortful than evaluating a price. Consequently, people may prefer looking at the price first to begin the decision process with less effort.

The design of our study confronted buyers and sellers with the identical problem of choosing money versus choosing a lottery ticket. The same choices evoked opposing pupillary reactivity in buyers and sellers. Pupils dilated to a larger degree when buyers chose the lottery ticket (i.e., buy) and when sellers chose money (i.e., sell). This diametric pattern may reflect the differential status quos of buyers and sellers as a result of their initial endowments. A decision that changes the status quo requires more evidence to be accumulated, which may take more mental effort and hence is accompanied by greater pupil dilation, consistent with prior findings of pupil dilation during perceptual (*40*) and financial (*24*) decision-making.

Pupillary reactivity, however, is likely to be an indicator but not a driver of evidence accumulation. Convergent findings from studies in primates and rodents suggest that pupil dilation during decision-making reflects, in part, increased activity within the noradrenergic arousal system [see (*52*) for a review]. We reason that activation of the arousal system energizes the process of evidence accumulation through increased vigilance, especially when a choice that changes the status quo is about to be made. Consequently, pupillary reactivity, as an index of underlying noradrenergic activity, accompanies the dynamics of evidence accumulation. The time taken to accumulate evidence is often proportional to the amount of mental effort or cognitive resources devoted during decision-making. It is therefore logical that response time is associated with decision-related pupillary reactivity. However, pupil dilation is unlikely to be a cause of response time but instead is an observable correlate of noradrenergic activity modulating the cognitive processes that support evidence accumulation over time.

On the basis of our findings, a typical failure of transaction due to the endowment effect can unfold over time in the following way. First, both the buyer and the seller are unmotivated by default to change their endowment unless a sufficiently good offer is provided. Once an offer is displayed, they iteratively inspect their initial holding and the potential gain, with priority given to their initial endowment, which, in turn, further magnifies its subjective value. Thus, many offers fail to motivate both the buyer and the seller to trade, resulting in a transaction failure. By contrast, a successful deal is achieved when the buyer and/or the seller attend to their potential gains more than they attend to their initial holdings, thereby updating their default beliefs and dilating their pupils.

Prior neuroimaging studies have identified distinct patterns of brain activation associated with the endowment effect. One study found that different valuation of goods by buyers and sellers tracked activity in the ventral striatum (*10*), a brain region in the reward circuit previously linked to loss aversion in financial decision-making (*53*). Other studies reported activation of the anterior insula, a brain region related to emotional arousal, associated with the reluctance to sell during trading (*9*, *11*). Our decomposition of valuation-related bias and response-related bias offers a potential framework to organize these findings. That is, valuation-related bias may be mediated, in part, by the ventral striatum, which computes the subjective value of goods differentially in buyers and sellers, whereas response-related bias may be mediated by anterior insula, which mediates physiological arousal in response to actions that may change the status quo.

Our computational modeling of the endowment effect was developed to exploit the design of our study using lottery tickets as goods. Relative to nonmonetary goods, the endowment effect for monetary lotteries, while reported previously in multiple studies [see (*4*) for a recent meta-analysis], tends to be weaker than the endowment effect observed for physical goods (*54*). Nevertheless, we chose to use lottery tickets as goods because there is a normative value for each lottery ticket—its expected value—which simplifies the computational model we applied to decompose potential biases underlying the endowment effect. Beyond our specific design and the limited sample size of our study, future studies should extend our experimental and analytical framework to accommodate nonmonetary goods and also characterize heterogeneity of the endowment effect in a larger, more representative population.

Last, our framework suggests separate interventions that can be used to mitigate endowment effects in markets. One way would be to intervene through manipulations of valuation-related bias via attention. For example, guiding attention of buyers and sellers to their potential gains instead of potential losses by selectively manipulating the salience of goods and prices, respectively, should increase the likelihood of a successful trade. Another way would be to intervene through response-related bias by, for example, setting the default options for buyers and sellers to be accepting offers rather than rejecting them. In this way, our study provides biologically and computationally grounded theoretical guidance for market designers and policy makers to improve the efficiency of free markets by introducing personalized interventions with potential to counteract friction induced by the endowment effect.

## MATERIALS AND METHODS

### Participants

The study was approved by the ethics committee of Zhejiang University. Sixty-four adults (33 female; 18 to 35 years old, mean ± SD = 23 ± 3 years) participated in the experiment and completed the trading task. Another three adults quit the experiment without starting the task because of failing to pass the calibration test of the eye tracker. All participants had normal or corrected-to-normal vision. Informed consent was obtained from all participants.

### Goods

Customized lottery tickets were prepared as the goods for trading in the experiment. Each lottery ticket had a visible face value printed on its front (e.g., ¥16), whereas the actual amount of money for which it could be redeemed was an integer randomly selected between 0 and its face value, which was printed on its back and covered by a scratch-off layer. Thus, the actual value of each lottery ticket was predetermined but hidden, and the expected value of a lottery ticket for participants was half of its face value.

### Endowment

Upon arrival, each participant was endowed with ¥55 cash, in addition to a stack of lottery tickets, which was randomly drawn by her/himself from two visually identical stacks by flipping a coin. Each stack consisted of 10 lottery tickets with face values ranging from ¥2 to ¥20 in ¥2 increments. Participants were briefed on how the actual value of the lottery tickets was determined. Consequently, the overall expected value of each stack of lottery tickets was also ¥55 [i.e., (¥2 + ¥4 + … + ¥20)/2]. In sum, each participant was endowed with both money and lottery tickets of equivalent value before the initiation of the trading task.

### Trading task

Each participant played the role of either a buyer or a seller in two separate blocks, with order counterbalanced across participants. On each trial, participants were presented with an offer, with the face value of a lottery ticket and a price displayed on the left and right to the center of a computer screen (48 cm by 30 cm, 1680 × 1050 pixels) (Fig. 1A). The positions of lottery ticket and price were fixed across the two roles for each participant but were counterbalanced across participants. In the buyer block, participants decided whether or not to buy the lottery ticket of the indicated face value atop the unendowed stack at the given price. In the seller block, participants decided whether or not to sell the lottery ticket of the indicated face value atop the endowed stack at the given price. In either block, the 10 lottery tickets of different face values were each presented 10 times, with the price varying from ¥1 to ¥10, in ¥1 increment. Thus, there were 100 unique offers in each block. The order of the buyer block and the seller block were counterbalanced across participants (see the “Order” section in Supplementary Text for analyses on potential order effect on the endowment effect). Participants made decisions for each offer by pressing one of two keys. Before the onset of each offer, a fixation cross was presented at the center of the screen with a varying duration of 2 to 4 s to maintain participants’ attention.

### Payment

All decisions were recorded but not executed immediately. Participants were informed that at the end of the trading task, their decision for one of all 200 offers would be randomly selected to be executed. If their decision for that offer was rejection, they exited the study with their original endowment, i.e., ¥55 cash plus the 10 lottery tickets in the endowed stack. If the offer was selected from the buyer block and their decision was acceptance, they paid the price and acquired the lottery ticket of the face value from the unendowed stack. If the offer was selected from the seller block and their decision was acceptance, they acquired the amount of money indicated by the price and gave up the lottery ticket of the face value from the endowed stack. All lotteries were redeemed to cash before the participants left the study. See the complete experimental instructions in the Supplementary Materials.

### Gaze and pupil data acquisition

Participants were seated about 60 cm in front of the screen in a dark and silent room. Gaze fixation and pupil diameter were sampled at 500 Hz using an infrared eye tracker (SensoMotoric Instruments). The eye tracker was synchronized with the stimulus presentation software (E-Prime).

### Processing of gaze data

Trials with fewer than 50% valid gaze position samples during the decision period (from offer onset to offset) were excluded from gaze analysis (1.13%). The boxes containing the amount of price or the face value of lottery ticket on the screen were identified as regions of interest (ROIs, each occupied about 14°). Total time spent looking at the price or the lottery ticket ROIs on each trial was calculated and referred to as “gaze duration” on price or lottery ticket. Gaze-lottery ratio was calculated for those trials that included at least one gaze fixation on either price or lottery ticket (99.00% of all valid trials). Heatmaps of gaze allocation probability were created by calculating the percent of gaze samples falling on each pixel within the ROIs on a trial and then averaged across trials (Fig. 3A).

### Processing of pupil data

Trials with fewer than 50% valid pupil samples in either the analysis time window (i.e., 0.5 s before offer onset to 1.5 s after decision) or the baseline time window (i.e., 0.5 to 0 s before offer onset) were excluded (1.77%). For simplicity, only the size of the left pupil was considered for analysis. Extreme or isolated pupil samples were excluded with respect to the standards proposed in (*55*). Missing or excluded samples were interpolated linearly. The pupil data were then resampled to 500 Hz, processed with a third-order Butterworth filter (low pass, 4 Hz), and *z*-scored for each block and each participant. For each trial, baseline correction was done by subtracting the mean value of the 0.5 s before offer onset from each value within the analytical time window. Decision-related pupil size was calculated as the mean pupil size in the time window from 0.5 s before to 1.5 s after decision.

### DDM modeling

In the DDM model, for each offer, decisions to buy and not to sell were both framed as a selection of the choice for the lottery ticket, and decisions to sell and not to buy were both framed as a selection of the choice for the amount of money indicated by the price. The decision process was assumed to be a noisy evidence-accumulation process unfolding between the upper boundary that governed the choice for the lottery ticket (denoted by 1) and the bottom boundary that governed the choice for the money (denoted by 0). For an offer with a price *P* and a lottery ticket of an expected value *L* (i.e., half of its face value), we assumed the drift rate *v* = *d*(*w* · *L* − *P*) + *b* + ɛ. Here, *w* denoted the weight assigned to the lottery ticket, which was assumed to be independent for buyer (*w*_b_) and seller (*w*_s_), and the logarithmic ratio between the two, ln(*w*_s_/*w*_b_), was calculated as DDM valuation bias. *d* was a scaling parameter that converted price and lottery ticket value to drift rate. *b* was an intercept that accounted for variance in choices and response times that was irrespective to the price and the expected value of the lottery ticket, which was also allowed to differ between roles (i.e., *b*_s_ and *b*_b_). ɛ indicated random noise in the diffusion process with a standard normal distribution, i.e., ɛ ~ *N* (0,1). The starting point of the evidence-accumulation process was denoted by *z*_b_ and *z*_s_ for the buyer and seller roles, respectively. DDM response bias was calculated as the difference between the two, i.e., *z*_s_ − *z*_b_. In addition, a parameter *a* was used to scale boundary separation or the distance of the two boundaries relative to noises, which was also allowed to vary by roles (i.e., *a*_s_ and *a*_b_). Last, nondecision time was captured by a parameter *t*.

We used the HDDM package (*56*) in Python to conduct hierarchical Bayesian estimation for our DDM. To allow our model to be implemented in the framework of HDDM, we converted *d*(*w* · *L* − *P*) in the drift rate to β1 · *L* + β2 · *P*. Consequently, *w* = −β1/β2. We ran a chain of 12,000 samples for the model and each of its reduced versions, with the first 2000 samples as burn-ins. DIC was calculated for model comparison. Means and 95% credible intervals of posterior distributions were reported for each parameter or the difference of two parameters when applicable.

To perform DDM-based prediction (fig. S2), for each participant and each offer, we calculated the probability density of the Wiener diffusion first passage time based on estimated parameters and then identified the response time and choice of the largest probability as our prediction. On the basis of predicted choices, we calculated the accuracy for each participant and then averaged for the group. On the basis of predicted response times, we calculated the Pearson correlation between predicted and actual response times for each participant and then averaged the correlations across the group.

### aDDM modeling

Building on our full DDM (DDM1), we tested a variant of aDDM in the framework of HDDM (*33*) by allowing the weight of lottery ticket to vary as a function of gaze allocation for both buyer and seller. Specifically, we assumed that when gaze was on the price, the weight of the lottery ticket was *w*_b,p_ and *w*_s,p_ for buyer and seller, respectively, and when gaze was on the lottery ticket, the weight was *w*_b,l_ and *w*_s,l_ for buyer and seller, respectively. For an offer with a price *P* and a lottery ticket of an expected value *L*, when the price was fixated, we assumed the drift rate, *v* ~ *w*_b,p_ · *L* − *P* for buyer and *v* ~ *w*_s,p_ · *L* − *P* for seller, and when the lottery ticket was fixated, we assumed the drift rate, *v* ~ *w*_b,l_ · *L* − *P* for buyer and *v* ~ *w*_s,l_ · *L* − *P* for seller. Assuming that the percent of time spent looking at the lottery ticket, or gaze-lottery ratio, was *GazeL*, and that for the price, or gaze-price ratio, was *GazeP* (i.e., 1 − *GazeL*), the mean drift rate for the offer was *v* ~ *GazeP* · *w*_b,p_ · *L* + *GazeL* · *w*_b,l_ · *L* – *P* for buyer and *v* ~ *GazeP* · *w*_s,p_ · *L* + *GazeL* · *w*_s,l_ · *L* − *P* for seller. We fit the aDDM in HDDM in a way similar to DDM except that only trials with valid eye positions were included (see the “Processing of gaze data” section).

### LM modeling

A variant of the LM (LM1) was built to serve as a time-flat version of our full DDM (DDM1). For an offer with a price *P* and a lottery ticket of an expected value *L*, we assumed the probability of choosing the lottery ticket *Prob* to vary by the linear combination of the price and the expected value of the lottery ticket in a way described by the following equations

$$Prob=\frac{1-\delta }{1+e^{-\mu (\lambda \cdot L-P)+\gamma }}+\delta \;\mathrm{if}\geq 0$$

$$Prob=\frac{1+\delta }{1+e^{-\mu (\lambda \cdot L-P)+\gamma }}\;\mathrm{if}<0$$

The weighting parameter λ here resembled the weighting parameter *w* in our DDM, denoting the relative weight of lottery ticket. By allowing the weighting parameter to be independent for buyer (λ_b_) and seller (λ_s_), we quantified LM valuation bias as the logarithmic ratio of the two, i.e., ln(λ_s_/λ_b_). The predisposition parameter δ here resembled the starting point *z* in our DDM. When δ is larger than zero, the probability of choosing the lottery ticket ranges from δ to 1, which denotes a predisposition toward choosing the lottery ticket. When δ is smaller than zero, the probability of choosing the lottery ticket ranges from 0 to 1 + δ, which denotes a predisposition toward choosing money. By allowing this parameter to be independent for buyer and seller, we quantified LM response bias as the difference between the two, i.e., δ_s_ − δ_b_. The logit parameter μ converted the subjective value derived from the contrast of the expected value of the lottery ticket and the price to choice probability. The parameter γ here resembled the parameter *b* in our DDM, denoting an intercept irrelevant to the price and the expected value of the lottery ticket, which is also allowed to be independent for buyer and seller. We used fmincon in Matlab to perform maximum likelihood estimation and obtained parameter estimates for each participant. BIC was calculated for model comparison.

To perform LM-based prediction (fig. S8), for each participant and each offer, we calculated the probability of choosing the lottery ticket using estimated parameters and made a binary prediction of the choice based on whether the probability was larger than 50% or not. We then computed prediction accuracy for each participant and averaged across the group.

### Regression analysis and *t* tests

For multiple regression analyses, each regressor was rescaled between 0 and 1. For mixed-level regressions, random effects of participants were included for the constant and all regressors. All *t* tests were two-tailed.

## Supplementary Materials

### This PDF file includes:

Supplementary Text Experimental Instructions Figs. S1 to S9 Tables S1 to S22
