# Supplementary material for: The art of the deal: Deciphering the endowment effect from traders’ eyes
Source: Sci Adv. 2023 Aug 23;9(34):eadf2115. doi: 10.1126/sciadv.adf2115 (PMC10446475; doi:10.1126/sciadv.adf2115)
Supplement: Supplementary file 1 — Supplementary Text Experimental Instructions Figs. S1 to S9 Tables S1 to S22 [file sciadv.adf2115_sm.pdf]

Science Advances
AAAS

Supplementary Materials for

The art of the deal: Deciphering the endowment effect from traders' eyes

Feng Sheng *et al.*

Corresponding author: Feng Sheng, fsheng@zju.edu.cn

Sci. Adv. **9**, eadf2115 (2023)
DOI: 10.1126/sciadv.adf2115

This PDF file includes:

Supplementary Text
Experimental Instructions
Figs. S1 to S9
Tables S1 to S22

Supplementary Text

Intercept. In our full DDM (DDM1), an intercept b is included in the drift rate to account for variance in choices and response times that is irrelevant to price and expected value of lottery ticket. Within our theoretical framework, the DDM intercept can either be understood as an additive component in valuation, which adds/subtracts a constant magnitude to/from the expected value of lottery ticket, or be understood as an inertial component of response tendency in drift rate, which constantly accumulates over time. The two possibilities are both captured by the intercept mathematically.

We found the intercept parameter was indispensable to account for variance observed in choices and response times because the DDM without it (DDM4, set b=0) performed much worse than the full DDM (△DIC=1454, see table S2). In addition, we allowed the intercept parameter to be independent for buyer (b_b) and seller (b_s) because the DDM that constrained the variation of intercept between buyer and seller (DDM5, set b_b=b_s) performed worse than the full DDM (△DIC=91, see table S2).

At the group level, we found a larger DDM intercept in sellers than in buyers (mean of difference=0.152, 95% credible interval: 0.013 to 0.294; see table S2 for details), suggesting either a larger fixed value added to lottery tickets by sellers or a larger inertia toward choosing lottery tickets during evidence accumulation in sellers.

Across individuals, we found the DDM intercept difference between seller and buyer (b_s-b_b) was correlated with neither DDM valuation bias (Pearson's r=-0.074, p=0.560) nor DDM response bias (Pearson's r=0.033, p=0.795). In addition, the DDM intercept difference between seller and buyer was not correlated with total gaze bias (Pearson's r=0.068, p=0.594), first gaze bias (Pearson's r=0.017, p=0.894) or pupillary reactivity bias (Pearson's r=0.179, p=0.157).

These findings, together, suggest that the variation of DDM intercept between seller and buyer explained a non-negligible proportion of variance in choices and response times that could not be accounted for by variation in either starting point or lottery weight. The DDM intercept difference between seller and buyer likely reflects a combination of valuation-related and response-related biases, which cannot be simply attributed to either of the two and cannot be easily disambiguated from gaze or pupillary response of traders.

Boundary Separation. The parameter of boundary separation a in our DDM scales the distance between the two response boundaries relative to noises. A larger boundary separation indicates a larger amount of evidence required to trigger either of the two choices, which often means a longer time taken to accumulate evidence. In our full DDM (DDM1), we allowed boundary separation to be independent for buyers and sellers because the DDM that constrained the variation of boundary separation between buyers and sellers (DDM6, set a_s=a_b) performed worse than the full DDM (△DIC=581, see table S2).

At the group level, we observed larger boundary separation in sellers than in buyers (mean of difference=0.340, 95% credible interval: 0.195 to 0.484; see table S2 for details), consistent with the longer time taken by sellers to make decisions. Relative to the role of a buyer, most participants should have fewer experiences being a seller in daily life, and may therefore be more indecisive when choosing whether or not to sell than when choosing whether or not to buy within the context of our study.

Across individuals, we found boundary separation difference between seller and buyer was not correlated with DDM valuation bias (Pearson's r=0.030, p=0.813), DDM response bias (Pearson's r=-0.060, p=0.635) or DDM intercept difference (Pearson's r=0.033, p=0.794), and was not associated with total gaze bias (Pearson's r=-0.020, p=0.873) or pupillary reactivity bias (Pearson's r=-0.014, p=0.911). We found a weak, but not statistically significant, correlation between boundary separation difference and first gaze bias (Pearson's r=0.219, p=0.082), suggesting participants who were more likely to look first at lottery tickets when acting as sellers than when acting as buyers, were also more indecisive when choosing whether to sell than when choosing whether to buy, although this was not statistically significant. See the First Gaze section below for more analyses and discussion on this finding.

First Gaze. In addition to individual difference analysis, we performed DDM modeling analysis to further clarify the relationship between first gaze and DDM parameters.

Motivated by the sign of the association between boundary separation difference and first gaze bias in individual difference analysis (see Boundary Separation section), we inferred that first gaze might account for part of variance explained by boundary separation in DDM. To test this possibility, based on our aDDM (aDDM1), we estimated an alternative model (aDDM2) that accommodated a potential modulatory effect of first gaze on boundary separation with a dummy variable f (1=first gaze on lottery ticket, 0=first gaze on price). This aDDM performed better than our baseline aDDM (△DIC=-52, see table S4). Importantly, we found a modulatory effect of first gaze on boundary separation that was significantly larger than zero (mean=0.183, 95% credible interval: 0.099 to 0.271; see table S4). That is, looking first at the lottery ticket was associated with a larger boundary separation. Notably, in this model (aDDM2), the role differences of the "static" boundary separation without first gaze modulation (i.e., a_s - a_b) remained significantly larger than zero (mean=0.328, 95% credible interval: 0.182 to 0.474; see table S4). We further examined whether this first gaze effect on boundary separation differed between roles by testing a third model (aDDM3) that allowed the first gaze parameter to be independent for buyers (f_b) and sellers (f_s). We found this model (aDDM3) did not perform better than the model (aDDM2) that set the first gaze parameter to be the same for buyers and sellers (△DIC=8) and the first gaze effects on boundary separation estimated by this model (aDDM3) were not different between sellers and buyers (mean of difference=-0.002, 95% credible interval: -0.150 to 0.140; see table S4 for details). These findings suggest that for both buyers and sellers, looking first at lottery tickets instead of prices might result in a more sluggish decision process. Relative to a price, a lottery ticket contains more information (i.e., the probability in addition to its face value). Thus, looking at lottery tickets first might require increased information processing, thus leading to a more deliberative decision process. This also explains why, across individuals, participants who were more likely to look first at lottery tickets in the seller block than in the buyer block showed larger boundary separation when acting as sellers than when acting as buyers (see Boundary Separation section).

Although our individual difference analysis found no correlation between first gaze bias and DDM response bias (fig. S4B, table S5), a recent study showed that the impact of early gaze biases, when not being modeled, could be erroneously attributed to starting point biases in DDM (*51*). That is, first gaze might account for variance explained by starting point. To test this possibility, based on our baseline aDDM (aDDM1), we tested a variant model (aDDM4) that accommodated potential modulatory effect of first gaze on starting point using a dummy variable j (1=first gaze on lottery ticket, 0=first gaze on price). We found this model performed slightly worse than the baseline aDDM (△DIC=1.4, table S4), without showing a significant first gaze effect on starting

point (mean=0.005, 95% credible interval: -0.008 to 0.017). We then tested another model (aDDM5) that allowed the first gaze effect on starting point to be independent for buyers (j_b) and sellers (j_s). This model (aDDM5) yielded negligible improvement in model fit relative to the baseline aDDM (aDDM1; △DIC=-0.06, table S4). Using this model, we observed no significant first-gaze effect on starting point in either sellers (mean=-0.004, 95% credible interval=-0.020 to 0.012) or buyers (mean=0.018, 95% credible interval=-0.000 to 0.038), although the lower bound of the 95% credible interval for buyers was just below zero. In this model, the "static" DDM response bias (z_s - z_b) without first gaze modulation remained significant (mean=0.057, 95% credible interval=0.038 to 0.078), with little change relative to that observed using the baseline aDDM (aDDM1; mean=0.053, 95% credible interval=0.034 to 0.073; see table S4). Across individuals, the Pearson correlation between the "static" DDM response biases estimated by this model (aDDM5) and the DDM response biases estimated by the baseline aDDM (aDDM1) reached 0.953 (p<0.001). Together, our modeling analysis yielded no evidence that first gaze could account for variance explained by starting point.

LM. The full logit model (LM1) we developed was a "time-flat" version of our DDM, which was built to merely account for variance in choices. Although this full LM performed worse than the LM that constrained LM response bias (LM3, table S9) and did not yield a significant LM response bias at the group-level, it provided a benchmark for our full DDM (DDM1) and allowed us to examine the extent to which individual differences in valuation-related bias and response-related bias were still identifiable when response time information was excluded from analysis.

Using this full LM (LM1), we found that LM valuation bias and LM response bias were not correlated with each other across individuals (fig. S7B, Pearson's r=0.034, p=0.788). Between LM and DDM, we found that LM valuation bias was correlated with DDM valuation bias (Pearson's r=0.807, p<0.001) but not with DDM response bias (Pearson's r=0.190, p=0.133). LM response bias was also correlated with DDM valuation bias (Pearson's r=0.255, p=0.042) and was not correlated with DDM response bias (Pearson's r=0.117, p=0.355).

Based on LM valuation bias and LM response bias, we can also segregate participants into four categories (fig. S7B). We found 64.10% of participants assigned to quadrant 1 by DDM were also assigned to the same quadrant by LM. This proportion was 33.33%, 25.00% and 33.33% for quadrants 2, 3 and 4, respectively. Overall, using LM only 51.56% of the participants in our study were assigned to the same quadrant they were assigned to using DDM. To understand the source of this discrepancy more deeply, we examined the categorization of valuation bias and response bias, respectively. We found DDM and LM similarly classified 82.81% of all participants when considering valuation bias alone, but similarly classified only 59.38% of all participants when considering response bias alone. Thus, inclusion of response times in modeling the endowment effect has a major potential impact on categorization of traders, especially with regard to response-related bias.

We next tested whether the two LM biases were reflected in total gaze bias, first gaze bias and pupillary reactivity bias by performing three separate regressions across individuals. We found total gaze bias was associated with LM valuation bias (fig. S9A, B=0.217, SE=0.062, t(61)=3.501, p<0.001) but not LM response bias (B=0.031, SE=0.057, t(61)=0.550, p=0.584; see the yellow column of table S10). Similarly, we found first gaze bias was associated with LM valuation bias (fig. S9B, B=0.342, SE=0.160, t(61)=2.134, p=0.037) but not LM response bias (B=0.134, SE=0.148, t(61)=0.905, p=0.369; see the yellow column of table S11). By contrast, we found pupillary reactivity bias was correlated with LM response bias (fig. S9C, B=0.423, SE=0.190,

t(61)=2.223, p=0.030) but not LM valuation bias (B=0.194, SE=0.206, t(61)=0.941, p=0.350; see the yellow column of table S12). Thus, LM retained the pattern of the selective association between eye activity and decision biases across individuals that was observed with DDM.

Notably, LM response bias and DDM response bias were not correlated with each other, but both of the two response biases were correlated with pupillary reactivity bias. To clarify this entangled relationship, we regressed pupillary reactivity bias simultaneously on DDM response bias and LM response bias. We found that pupillary reactivity bias was predicted by both DDM response bias (B=0.333, SE=0.138, t(61)=2.408, p=0.019) and LM response bias (B=0.377, SE=0.184, t(61)=2.045, p=0.045; see the yellow column of table S13 for details). That is, DDM response bias and LM response bias accounted for separable variance in pupillary reactivity bias across individuals.

Similar to the intercept *b* in our full DDM, we also included an intercept γ in the full LM and allowed it to be independent for buyers (γ_b) and sellers (γ_s). At the group-level, we observed no significant difference in intercept between buyers (M±SD=-5.176±13.006) and sellers (M±SD=-6.522±3.777; t(63)=0.855, p=0.396; see table S9 for details). Accordingly, we found that the full LM performed worse than the LM that constrained the variation of intercept between buyers and sellers (LM5, γ_b=γ_s; △BIC=71), although the full LM performed better than the LM without an intercept (LM4, set γ=0; △BIC=-1239).

Across individuals, we found LM intercept difference between seller and buyer (γ_s-γ_b) was positively correlated with LM valuation bias (Pearson's r=0.648, p<0.001) but not LM response bias (Pearson's r=-0.077, p=0.545). Between LM and DDM, we found LM intercept difference was positively correlated with DDM valuation bias (Pearson's r=0.250, p=0.046) but not DDM response bias (Pearson's r=0.018, p=0.886). In addition, LM intercept difference was negatively correlated with DDM intercept difference (Pearson's r=-0.315, p=0.011).

We also examined whether LM intercept difference between seller and buyer was reflected in eye activity. We found LM intercept difference was correlated with neither total gaze bias (Pearson's r=0.147, p=0.247) nor first gaze bias (Pearson's r=0.043, p=0.737). We observed a weak, but not significant, association between LM intercept difference and pupillary reactivity bias (Pearson's r=-0.210, p=0.096). Together, similar to DDM intercept difference, we found LM intercept difference between seller and buyer was hardly reflected in gaze or pupillary reactivity.

Order. In our study, half of the participants played the role of buyer in the first block and the role of seller in the second block, while the other half of them played the two roles in the opposite order. This design allowed us to estimate the endowment effect not only in a within-subject manner but also in a between-subject manner, and enabled us to explore potential order effect and learning effect. Specifically, we could assess potential within-subject endowment effect by coupling the buyer role and the seller role played by each participant and test whether the effect differed between the group of participants playing the buyer role first (i.e., buyer-first group) and the group of participants playing the seller role first (i.e., seller-first group). That is, testing potential order effect. Alternatively, we could assess potential between-subject endowment effect by coupling the buyers and the sellers either within the first block or within the second block and test whether the effect differed in the second block relative to the first block. That is, testing potential learning effect.

We first examined how order impacted the probability of choosing lottery tickets. We found that relative to buyers, sellers were more likely to choose lottery tickets both in the buyer-first group and in the seller-first group, and also both in the first block and in the second block (table

S14, all ps<0.002). To explore potential order effect and learning effect, we applied a 2 (Role: seller vs. buyer) by 2 (Group: seller first vs. buyer first) analysis of variance (ANOVA) to the probability of choosing lottery tickets. Order effect was quantified by the interaction between Role and Group, which was not significant (F(1,62)=0.087, p=0.769). Learning effect was captured by the main effect of Group, which might not be obvious so we explain further. Assume the endowment effect is smaller in the second block with buyers choosing more lottery tickets and sellers choosing fewer lottery tickets—a learning effect. This would manifest in a pattern in which, relative to the buyer-first group, the seller-first group chose more lottery tickets when acting as buyers (in the second block) and also chose more lottery tickets when acting as sellers (in the first block), resulting in more lottery tickets chosen by the seller-first group. In our sample, the main effect of Group was not significant (F(1,62)=0.437, p=0.511), suggesting no learning effect.

We then conducted similar analysis on response time. We calculated the differential response time between choosing lottery tickets and choosing money to reversely index the tendency to choose lottery tickets over money. We found the differential response time between choosing lottery tickets and choosing money was larger in buyers than in sellers both in the buyer-first group and in the seller-first group, and also both in the first block and in the second block (table S15, all ps<0.019). Again, a 2 (Role) by 2 (Group) ANOVA yielded neither a significant interaction effect (F(1,62)=1.672, p=0.201) nor a significant main effect of Group (F(1,62)=0.216, p=0.644), indicating absence of order effect and learning effect indexed by response time.

We next applied this set of analyses to parameter estimates of our full DDM (DDM1). We found relative to buyers, sellers assigned a larger weight to lottery tickets both in the buyer-first group and in the seller-first group, and also both in the first block and in the second block (table S16, all ps<0.027). A 2 (Role) by 2 (Group) ANOVA showed neither an interaction effect (F(1,62)=1.321, p=0.255) nor a main effect of Group (F(1.62)=0.118, p=0.732). Similar to lottery weight, we found relative to buyers, sellers showed a higher starting point (i.e., closer to the boundary to choose lottery ticket) both in the buyer-first group and in the seller-first group, and also both in the first block and in the second block (table S17, all ps<0.002). A 2 (Role) by 2 (Group) ANOVA identified a marginally significant interaction effect (F(1,62)=3.610, p=0.062). That is, the starting point difference between seller and buyer, or DDM response bias, tended to be larger in the seller-first group (M=0.063) than in the buyer-first group (M=0.041), suggesting a minor order effect. The main effect of Group was not significant (F(1,62)=0.164, p=0.687), suggesting no learning effect. See table 17 for details.

We also examined how order impacted DDM intercept and boundary separation. We found relative to the intercept of buyer, the intercept of seller was significantly larger in the buyer-first group (table 18, t(31)=2.864, p=0.007), marginally larger in the seller-first group (t(31)=1.945, p=0.061), marginally larger in the first block (t(62)=1.834, p=0.071) and not larger in the second block (t(62)=0.561, p=0.577). Neither a significant interaction effect (F(1,62)=0.540, p=0.465) nor a main effect of Group (F(1,62)=0.476, p=0.493) was found with a 2 (Role) by 2 (Group) ANOVA. We also found that relative to the boundary separation of buyer, the boundary separation of seller was significantly larger both in the buyer-first group and in the seller-first group (table 19, both ps<0.002), and was not larger in the first block (t(62)=0.597, p=0.553) but significantly larger in the second block (t(62)=2.675, p=0.010). Again, a 2 (Role) by 2 (Group) ANOVA revealed neither a significant interaction effect (F(1,62)=1.861, p=0.177) nor a significant main effect of Group (F(1,62)=1.382, p=0.244).

We then examined the impact of order on gaze allocation. We found relative to buyer, the gaze-lottery ratio of seller was not larger in the buyer-first group (table 20, t(31)=1.016, p=0.317)

but marginally larger in the seller-first group ($t(31)=1.866$, $p=0.072$), and not larger either in the first block or in the second block (both $ps>0.462$). A 2 (Role) by 2 (Group) ANOVA showed neither a significant interaction effect ($F(1,62)=0.024$, $p=0.876$) nor a significant main effect of Group ($F(1,62)=0.002$, $p=0.969$). Relative to buyers, the first-gaze lottery probability of sellers was larger both in the buyer-first group and in the seller-first group (table 21, both $ps<0.009$), and larger in the first block ($t(62)=4.198$, $p<0.001$) but not larger in the second block ($t(62)=1.227$, $p=0.224$). A 2 (Role) by 2 (Group) ANOVA revealed a small, but insignificant, interaction effect ($F(1,62)=2.893$, $p=0.094$). That is, the difference of first-gaze lottery probability between seller and buyer, or first gaze bias, tended to be larger in the seller-first group (M=0.191) than in the buyer-first group (M=0.105), suggesting a minor order effect. We also found a small, but insignificant, main effect of Group ($F(1,62)=2.940$, $p=0.091$). That is, first gaze bias tended to be larger in the first block (M=0.231) than in the second block (M=0.066), suggesting a minor learning effect. See table 21 for details.

We also analyzed the impact of order on pupillary reactivity. We calculated the differential decision-related pupil size between choosing lottery ticket and choosing money to index potential asymmetric pupillary reactivity to different choices. We found the differential decision-related pupil size between choosing lottery ticket and choosing money was larger in buyers than in sellers both in the buyer-first group and in the seller-first group, and also both in the first block and in the second block (table S22, all $ps<0.003$). A 2 (Role) by 2 (Group) ANOVA identified neither a significant interaction effect ($F(1,62)=0.106$, $p=0.746$) nor a significant main effect of Group ($F(1,62)=0.268$, $p=0.607$).

Finally, we examined whether our findings based on individual difference analysis changed when order was included as a control variable (0=buyer first, 1=seller first). We found all findings held (see the last columns of table S3, table S5, table S6, table S8, table S10, table S11, table S12 and table S13).

Together, we found no statistically significant order effect or learning effect across different indices of the endowment effect. DDM response bias tended to be larger in the seller-first group. First gaze bias tended to be larger in the seller-first group and in the first block.

Experimental Instructions

The experimental instructions consisted of three parts. The first part was the general instruction given to participants to introduce the cover story and to allocate the endowment. The other two parts were instructions delivered at the beginning of the buyer block and the beginning of the seller block, respectively. The instructions were delivered in Chinese and here were translated into English.

A quiz was included in each of the three parts to test participants' understanding. When a participant failed to correctly answer all questions in a quiz, the experimenter guided the participant to reread the specific contents and asked the participant to redo the quiz. Thus, no participants were excluded by quizzes.

In the study, the instructions were shown as well as read out to participants. The contents in parentheses below were notes for the experimenter, not shown to participants. In the quizzes below, the first answer for each question was the correct one. The actual order of the answers for each question was randomized when the experiment was carried out.

(General instruction)

Welcome to the study today. This study is about trading. You will play the role of either **a buyer** or **a seller** at different blocks of the study.

Goods

In the study, the goods for trading are customized lottery tickets. Here is an example.

(show a lottery ticket of a ¥16 face value)

Each lottery ticket has a face value printed on its front, for example, ¥16, which indicates the maximal amount of money for which the lottery ticket can be redeemed. The actual value of a lottery ticket will be equal to or smaller than its face value.

On the back of each lottery ticket, there is a number that is covered by a scratch-off layer, which indicates the actual amount of money for which the lottery ticket can be redeemed. The number was **randomly** selected from all integers **between 0 and the face value of the lottery ticket** when it was made. Thus, the number on the back of a lottery ticket with a ¥16 face value can be **any** integer between 0 and 16 (including 0 and 16). If the number is 5, the lottery ticket can be redeemed for ¥ 5. If the number is 12, the lottery ticket can be redeemed for ¥ 12. If the number is 0, the lottery ticket can be redeemed for nothing. If the number is 16, the lottery ticket can be redeemed for ¥16. The number on the back will not larger than the face value of a lottery ticket on its front. Thus, the number on the back of a lottery ticket with a ¥16 face value will not larger than 16. The number on the back of a lottery ticket with a ¥4 face value will not larger than 4.

Note, the number printed on the back of a lottery ticket was selected **randomly** from all integers **between 0 and its face value** when the ticket was made. Thus, two lottery tickets of the same face value may have different numbers on its back. In other words, two lottery tickets of the same face value may be redeemed for different amounts of money.

For example, here are two lottery tickets both with a ¥16 face value. Please scratch off the layer on the back of each lottery ticket and check the printed number.

(hand the participant two lottery tickets of a ¥16 face value and guide the participant to scratch off the layers)

Thus, before scratching offer the layer on the back of a lottery ticket, you can only know the range of the amount of money for which the lottery ticket can be redeemed, but not the exact amount.

Initial asset

There are two stacks of lottery tickets on the table. Each stack consists of 10 lottery tickets, with their face values ranging from ¥2 to ¥20. That is, ¥2, ¥4, ¥6, ¥8, ¥10, ¥12, ¥14, ¥16, ¥18 and ¥20. In the study, one stack of lottery tickets is yours, and the other is not yours. Which of the two stacks is yours will be determined by a coin flip. If the coin lands with heads, the left stack of lottery tickets is yours, and if the coin lands with tails, the right is yours. Now, please flip this coin.

(give the participant a coin)

(hand the participant the stack of lottery tickets based on the outcome of the coin flip)

Now please hold this stack of lottery tickets. They are your asset. In addition, you have ¥55 cash in the study. Please hold it, too.

(hand the participant ¥55 cash)

Together, before performing any task, your initial asset includes **the 10 lottery tickets plus ¥55 cash**. Please count.

(guide the participant to count)

Please hold your asset.

Task

In the study, you will play the role of **a buyer** and the role of **a seller** in two separate blocks, respectively.

When acting as a buyer, you have the opportunity to buy **the lottery tickets in the stack left on the table**. You will make many decisions of whether or not to buy in many rounds. In each round,

the computer screen will display an offer with a price and the face value of a lottery ticket. You need to decide whether or not to buy the lottery ticket at the displayed price.

When acting as a seller, you have the opportunity to sell **the lottery tickets you have**. You will make many decisions of whether or not to sell in many rounds. In each round, the computer screen will display an offer with a price and the face value of a lottery ticket. You need to decide whether or not to sell the lottery ticket at the displayed price.

Final asset

In the study, your choice in each round will be recorded, but will not be implemented immediately.

After you complete all rounds of choices, the computer will **randomly** select **one round** of your choice and implement it.

If you are a buyer in the selected round and you choose not to buy, you simply keep all of your initial asset.

If you are a buyer in the selected round and you choose to buy, you pay the displayed price and get the lottery ticket of that face value from the stack of lottery tickets on the table.

If you are a seller in the selected round and you choose not to sell, you simply keep all of your initial asset.

If you are a seller in the selected round and you choose to sell, you sell the lottery ticket of that face value and get the amount of money equal to the displayed price.

Note, you will make many rounds of choices, but only **one** of the choices will be **randomly** selected to be implemented in the end. Here, please pay attention to two points.

First, **randomly**. All of your choices have the equal chance to be selected in the end. That is, any of your choices can be the one that finally impacts your asset.

Second, **one**. Only one of all your choices will be selected to be implemented. That is, your final asset will be impacted just by the selected choice but not by the other choices. Thus, each of your choices is independent.

Payment

By the end of the study, the layers on the backs of the lottery tickets you have will be scratched off, and all of the lottery tickets you have will be redeemed for the amounts of money equal to the numbers printed on their backs. Thus, you will leave the study with **the amount of cash you have after the trading task** plus **the amount of money you receive by redeeming the lottery tickets you have**.

(General quiz)

Quiz

(the order of the answers for each question is randomized)

Below is a quiz that tests your understanding of the task. Please choose the best answer for each question.

1. What is the amount of money that a lottery ticket can be redeemed for?
Its face value
The amount of money equal to the number on its back

2. What can be the number on the back of a lottery ticket?
Any integer between 0 and its face value
It can only be its face value
It can only be half of its face value

3. For a lottery ticket with a face value ¥14, which of the following statement is true?
The number on the back of the lottery ticket is equally likely to be 0, 5, 10 or 14.
The number on the back of the lottery ticket is most likely to be 0.
The number on the back of the lottery ticket is most likely to be 5.
The number on the back of the lottery ticket is most likely to be 10.
The number on the back of the lottery ticket is most likely to be 14.

4. What are the face values of the 10 lottery tickets you have?
¥2, ¥4, ¥6, ¥8, ¥10, ¥12, ¥14, ¥16, ¥18 and ¥20, respectively
¥2, ¥2, ¥2, ¥2, ¥2, ¥2, ¥2, ¥2, ¥2 and ¥2, respectively
¥10, ¥10, ¥10, ¥10, ¥10, ¥10, ¥10, ¥10, ¥10 and ¥10, respectively
¥20, ¥20, ¥20, ¥20, ¥20, ¥20, ¥20, ¥20, ¥20 and ¥20, respectively

5. What are the face values of the 10 lottery tickets left on the table?
¥2, ¥4, ¥6, ¥8, ¥10, ¥12, ¥14, ¥16, ¥18 and ¥20, respectively
¥2, ¥2, ¥2, ¥2, ¥2, ¥2, ¥2, ¥2, ¥2 and ¥2, respectively
¥10, ¥10, ¥10, ¥10, ¥10, ¥10, ¥10, ¥10, ¥10 and ¥10, respectively
¥20, ¥20, ¥20, ¥20, ¥20, ¥20, ¥20, ¥20, ¥20 and ¥20, respectively

6. There are a lottery ticket of a ¥16 face value you have and a lottery ticket of the same face value in the stack left on the table. Are the numbers on the backs of the two lottery tickets the same?

Not necessarily the same
Must be the same

7. How much is your initial asset?
¥55 cash plus 10 lottery tickets of face values ranging from ¥2 to ¥20
¥55 cash
10 lottery tickets of face values ranging from ¥2 to ¥20

8. Will each of your choices be implemented?
No, one round of your choice will be randomly selected to be implemented
No, one round of your choice when you act as a buyer will be randomly selected to be implemented
No, one round of your choice when you act as a seller will be randomly selected to be implemented
Yes

9. Will the selection of the choice for implementation be impacted by what choices you make?
No, every choice has the equal chance to be selected for implementation, regardless of what choice it is
Yes, the selected choice can only be a choice of buying a lottery ticket
Yes, the selected choice can only be a choice of not buying a lottery ticket
Yes, the selected choice can only be a choice of selling a lottery ticket
Yes, the selected choice can only be a choice of not selling a lottery ticket

10. How much will your final asset be?
The cash plus the lottery tickets you have after the implementation of the selected choice
The cash you have after the implementation of the selected choice
The lottery tickets you have after the implementation of the selected choice
The initial asset

(Instruction for buyer)

Buyer

In this block, you will play as a buyer. You have the opportunity to buy **those lottery tickets in the stack left on the table** with **the cash** you have.

You will make 100 rounds of choices. In each round, the computer screen will display an offer with a price and the face value of a lottery ticket. You need to press one of two keys to indicate whether or not to buy the lottery ticket at the displayed price.

If you choose to buy, press the ↑ key, and if you choose not to buy, press the ↓ key.

(the association between key presses and choices is counterbalanced between participants.)

For example, in the offer displayed below, you need to choose whether or not to buy a lottery ticket of a ¥16 face value at the price ¥7.

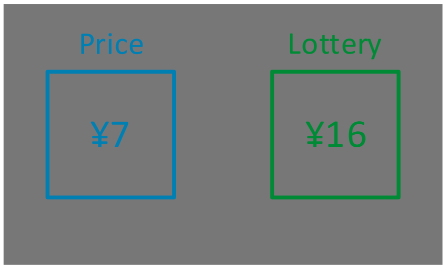

In the study, each of your choices will be recorded, but not implemented immediately.

After you make your choices in both buyer and seller blocks, the computer will randomly select one choice and implement it.

Assume the selected choice is the one you make for the offer displayed above when you act as a buyer.

If your choice is not to buy the lottery ticket, you simply keep your initial asset.

If your choice is to buy the lottery ticket, you will spend **¥7 out of the ¥55 cash you have** and get **the lottery ticket of a ¥16 face value from the stack on the table**.

When you do the task, please keep your head stable, so that the eye-tracker can monitor your eye activity.

Before each offer, the computer screen will display a cross fixation. At that time, please look at it rather than anywhere else.

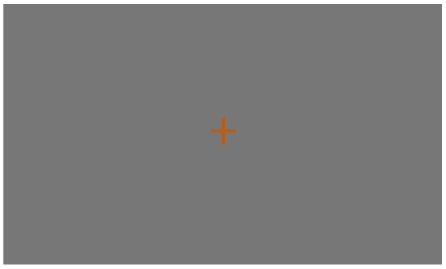

Once the computer screen displays an offer, you can look freely.

If you have any question, please ask the experimenter.

（Quiz for buyer）

Quiz

(the order of the answers for each question is randomized)

Below is a quiz that tests your understanding of the task in this block. Please choose the best answer for each question.

1. What is your role in this block?
Buyer
Seller

2. How can you indicate your choice in each round?
To buy, press ↑ and not to buy, press ↓
To buy, press ↓ and not to buy, press ↑

3. After you make a choice
The computer will record your choice, but will not implement it immediately
The computer will implement your choice immediately

4、 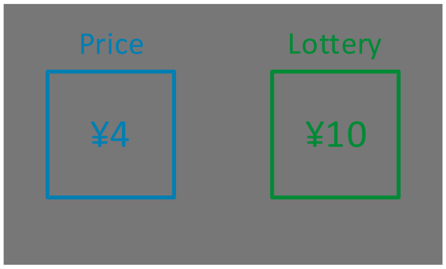

Assume the choice selected to be implemented in the end is the one you make for the offer displayed above when you act as a buyer. If your choice is not to buy the lottery ticket, what will happen to your asset?
You will simply keep your initial asset.
You will spend ¥4 out of the ¥55 cash you have and get the lottery ticket of a ¥10 face value from the stack on the table.
You will spend ¥4 out of the ¥55 cash you have.
You will get the lottery ticket of a ¥10 face value from the stack on the table.

5、 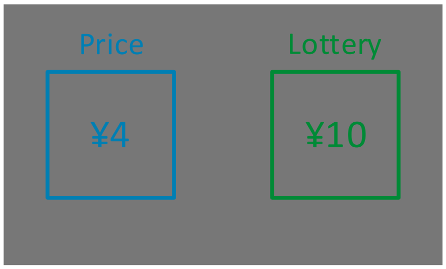

Assume the choice selected to be implemented in the end is the one you make for the offer displayed above when you act as a buyer. If your choice is to buy the lottery ticket, what will happen to your asset?
You will spend ¥4 out of the ¥55 cash you have and get the lottery ticket of a ¥10 face value from the stack on the table.
You will simply keep your initial asset.
You will spend ¥4 out of the ¥55 cash you have.
You will get the lottery ticket of a ¥10 face value from the stack on the table.

6. 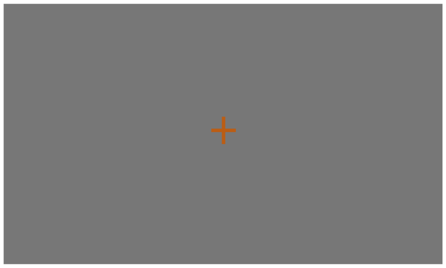
When a cross fixation is displayed on the computer screen
You should look at it.
You can look freely.

7. When you do the task
You need to keep your head stable.
You can move your head at will.

(Instruction for seller)

Seller

In this block, you will play as a seller. You have the opportunity to sell **the lottery tickets you have** and get money.

You will make 100 rounds of choices. In each round, the computer screen will display an offer with a price and the face value of a lottery ticket. You need to press one of two keys to indicate whether or not to sell the lottery ticket at the displayed price.

If you choose to sell, press the ↑ key, and if you choose not to sell, press the ↓ key.

(the association between key presses and choices is counterbalanced between participants.)

For example, in the offer displayed below, you need to choose whether or not to sell a lottery ticket of a ¥16 face value at the price ¥7.

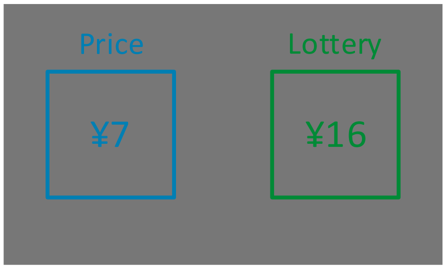

In the study, each of your choices will be recorded, but not implemented immediately.

After you make your choices in both buyer and seller blocks, the computer will randomly select one choice and implement it.

Assume the selected choice is the one you make for the offer displayed above when you act as a seller.

If your choice is not to sell the lottery ticket, you simply keep your initial asset.

If your choice is to sell the lottery ticket, you will sell **the lottery ticket of a ¥16 face value you have** and get **¥7**.

When you do the task, please keep your head stable, so that the eye-tracker can monitor your eye activity.

Before each offer, the computer screen will display a cross fixation. At that time, please look at it rather than anywhere else.

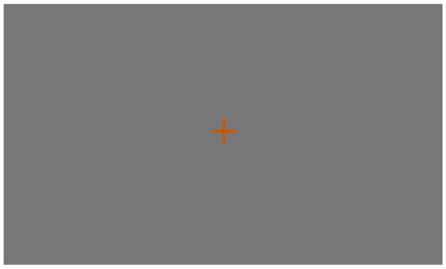

Once the computer screen displays an offer, you can look freely.

If you have any question, please ask the experimenter.

(*Quiz for seller*)

Quiz

(the order of the answers for each question is randomized)

Below is a quiz that tests your understanding of the task in this block. Please choose the best answer for each question.

1. What is your role in this block?
Seller
Buyer

2. How can you indicate your choice in each round?
To sell, press ↑ and not to sell, press ↓
To sell, press ↓ and not to sell, press ↑

3. After you make a choice
The computer will record your choice, but will not implement it immediately
The computer will implement your choice immediately

4、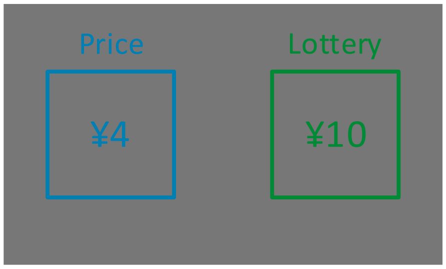

Assume the choice selected to be implemented in the end is the one you make for the offer displayed above when you act as a seller. If your choice is not to sell the lottery ticket, what will happen to your asset?
You will simply keep your initial asset.
You will sell the lottery ticket of a ¥10 face value you have and get ¥4 cash.
You will sell the lottery ticket of a ¥10 face value you have.
You will get ¥4 cash.

5、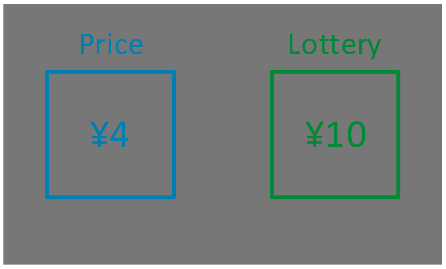

Assume the choice selected to be implemented in the end is the one you make for the offer displayed above when you act as a seller. If your choice is to sell the lottery ticket, what will happen to your asset?

You will sell the lottery ticket of a ¥10 face value you have and get ¥4 cash.

You will simply keep your initial asset.

You will sell the lottery ticket of a ¥10 face value you have.

You will get ¥4 cash.

6. 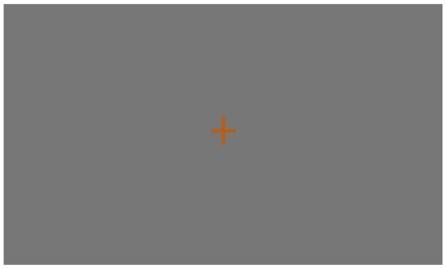

When a cross fixation is displayed on the computer screen

You should look at it.

You can look freely.

7. When you do the task

You need to keep your head stable.

You can move your head at will.

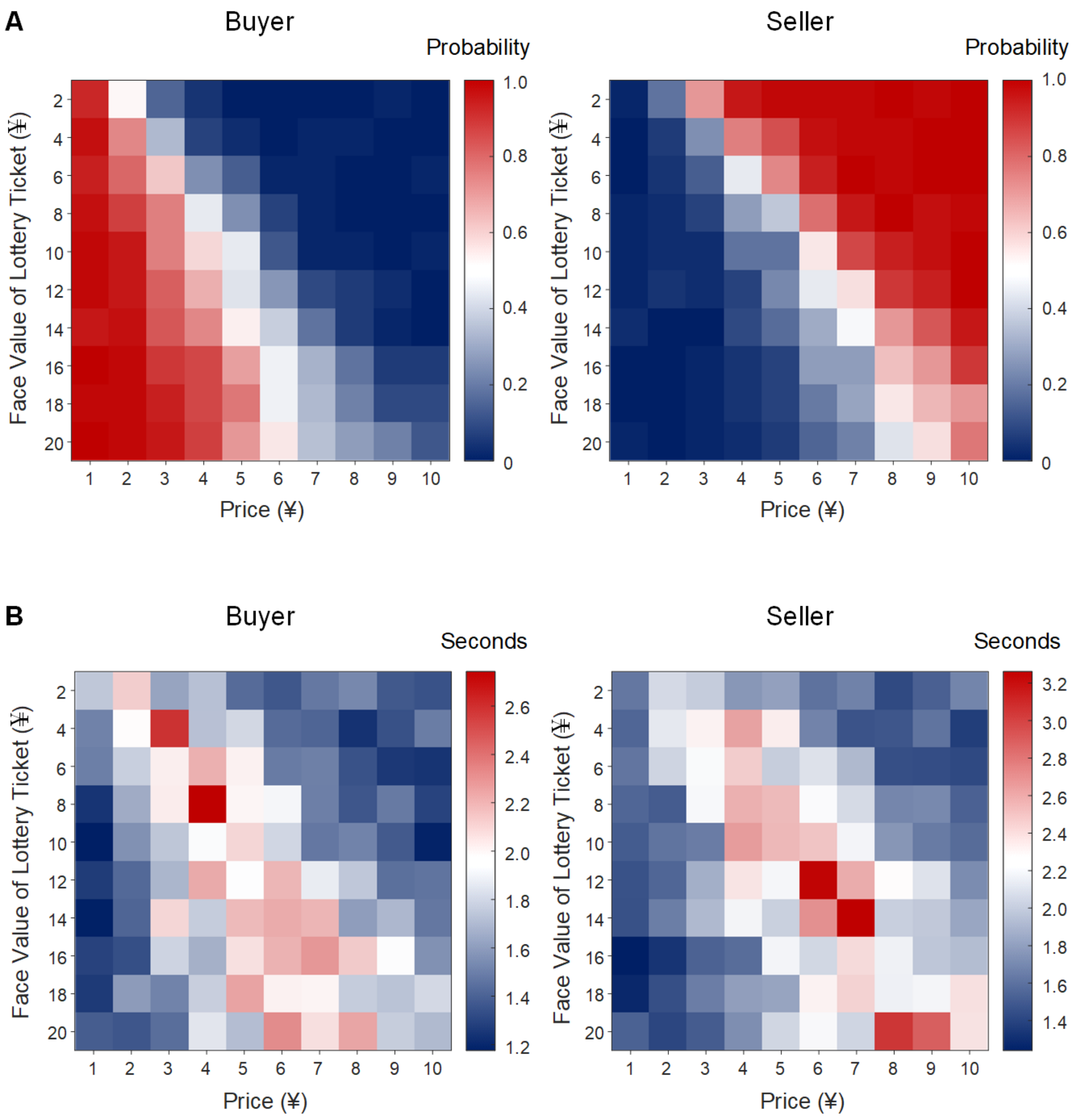

fig. S1. Probability of acceptance (A) and response time (B) across offers when participants acted as buyers and sellers, respectively. Each cell denotes an offer. The x axes indicate prices and the y axes indicate face values of lottery tickets.

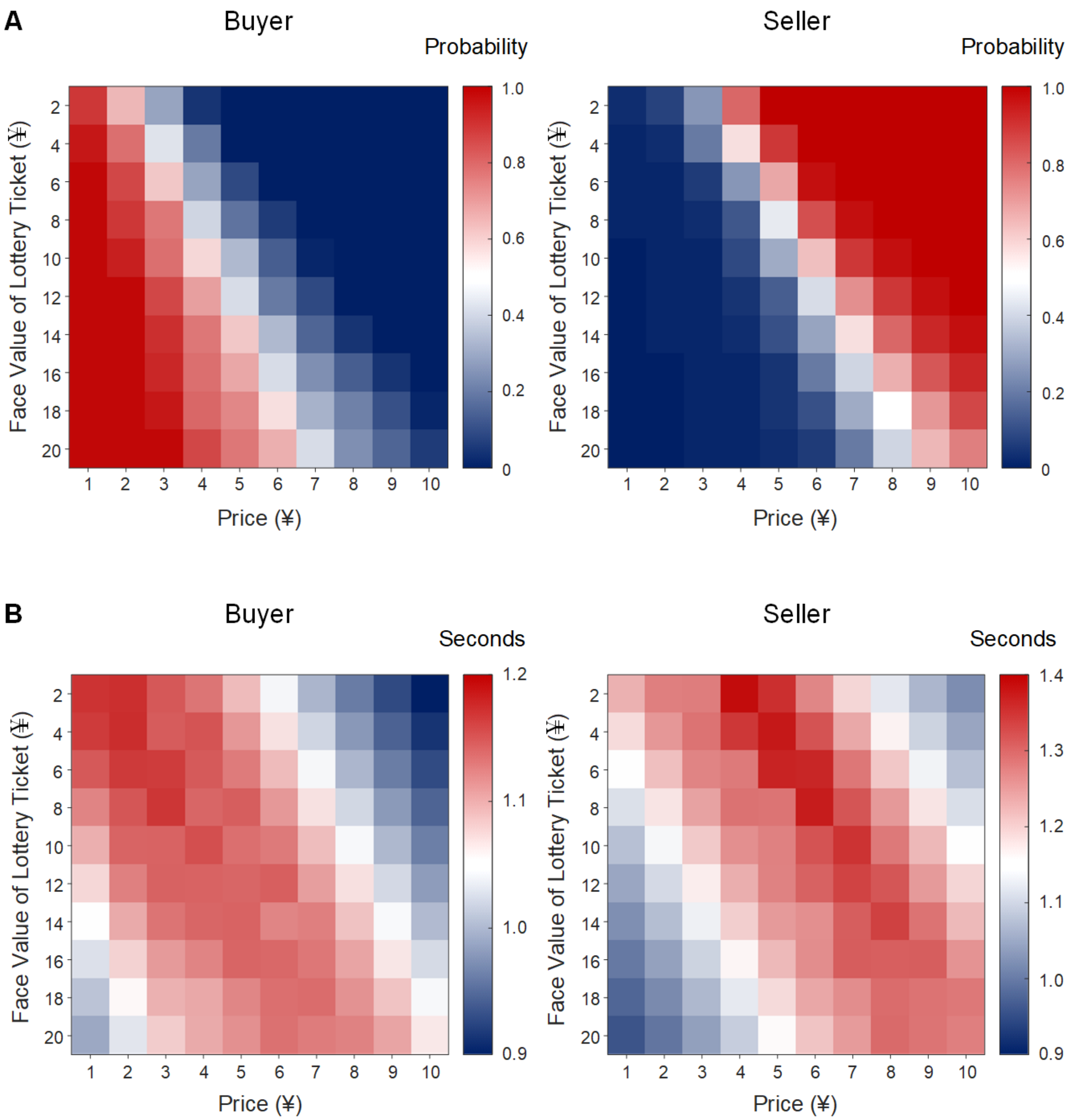

fig. S2. DDM-predicted probability of acceptance (A) and response time (B) across offers when participants acted as buyers and sellers, respectively. Each cell denotes an offer. The x axes indicate prices and the y axes indicate face values of lottery tickets.

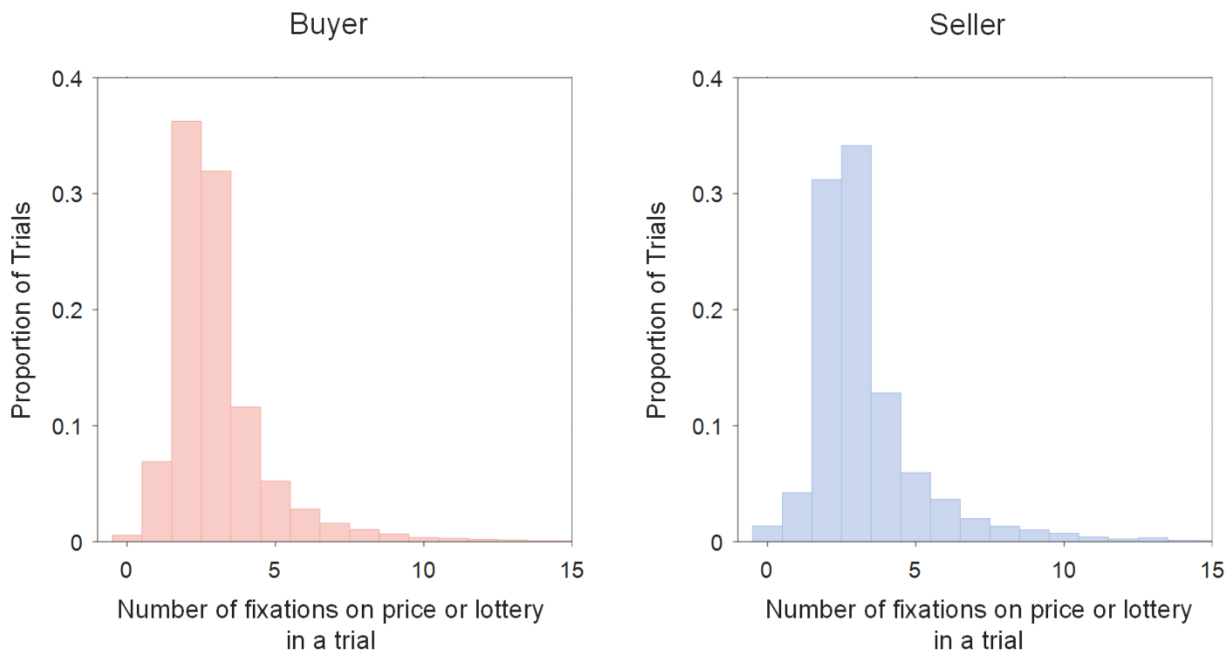

fig. S3. Histograms of the number of gaze fixations per trial when participants acted as buyers (left) and sellers (right), respectively.

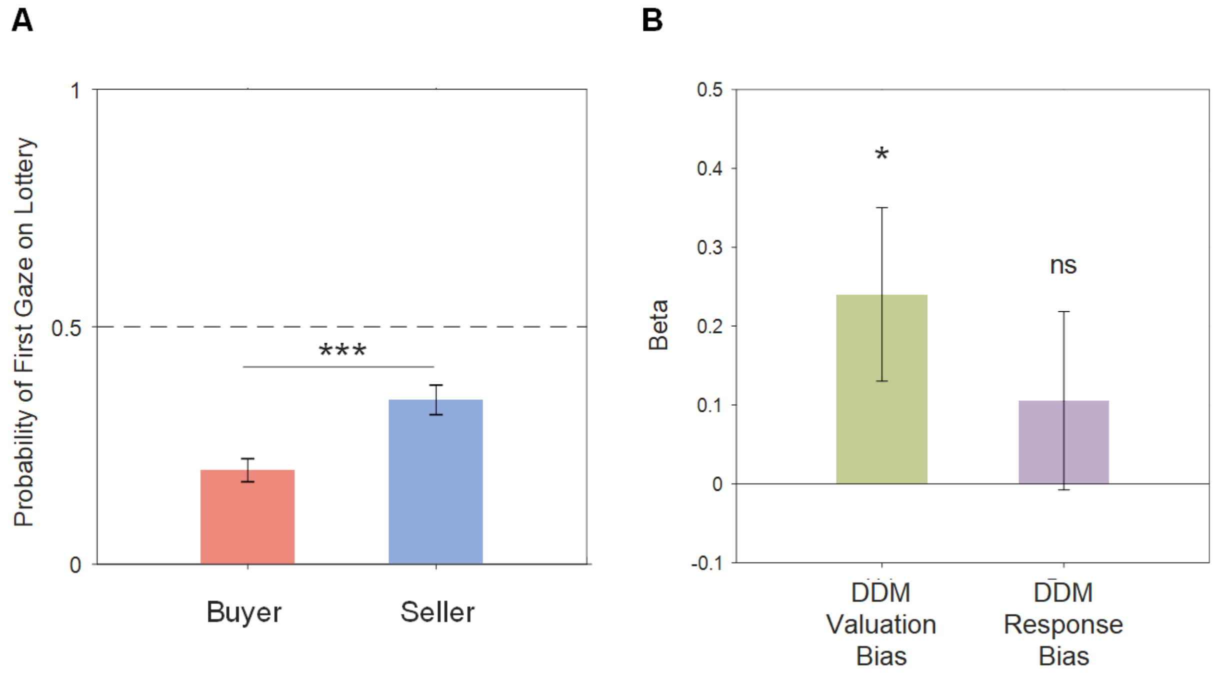

fig. S4. First Gaze. (A) Probability of first gaze on lottery tickets when participants acted as buyers and sellers, respectively. (B) First gaze bias predicted by DDM valuation bias and DDM response bias. *: p<0.05. ***: p<0.001. ns: nonsignificant.

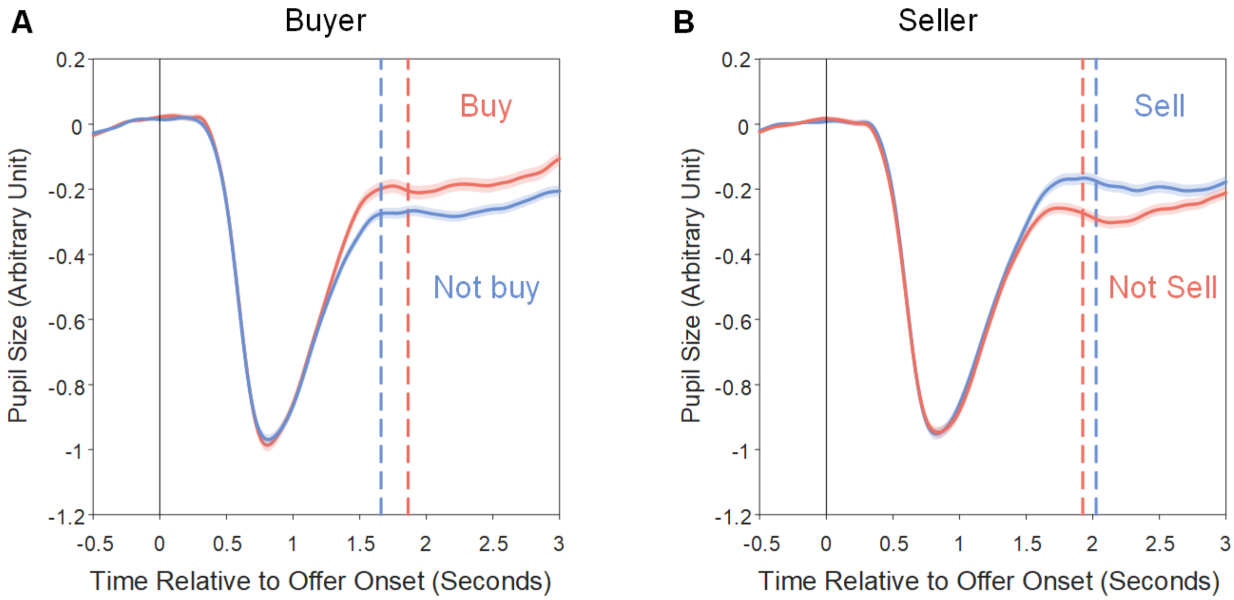

fig. S5. The time courses of pupil size aligned to offer onset. The dashed vertical lines indicate mean response time. Shaded areas indicate standard errors.

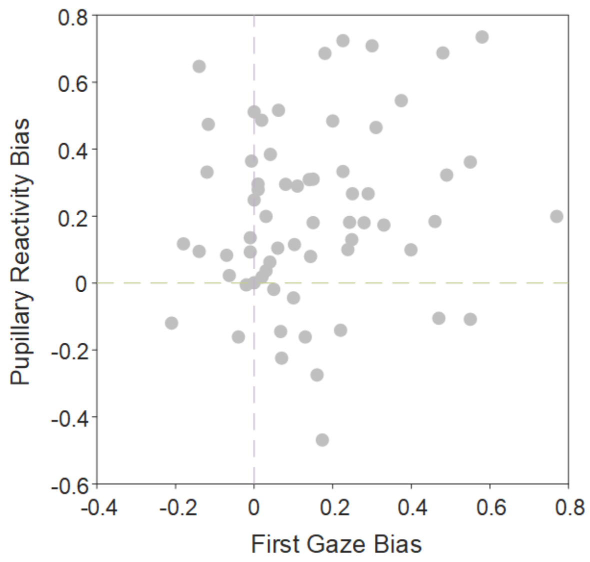

fig. S6. The scatter plot of first gaze bias and pupillary reactivity bias across individuals.

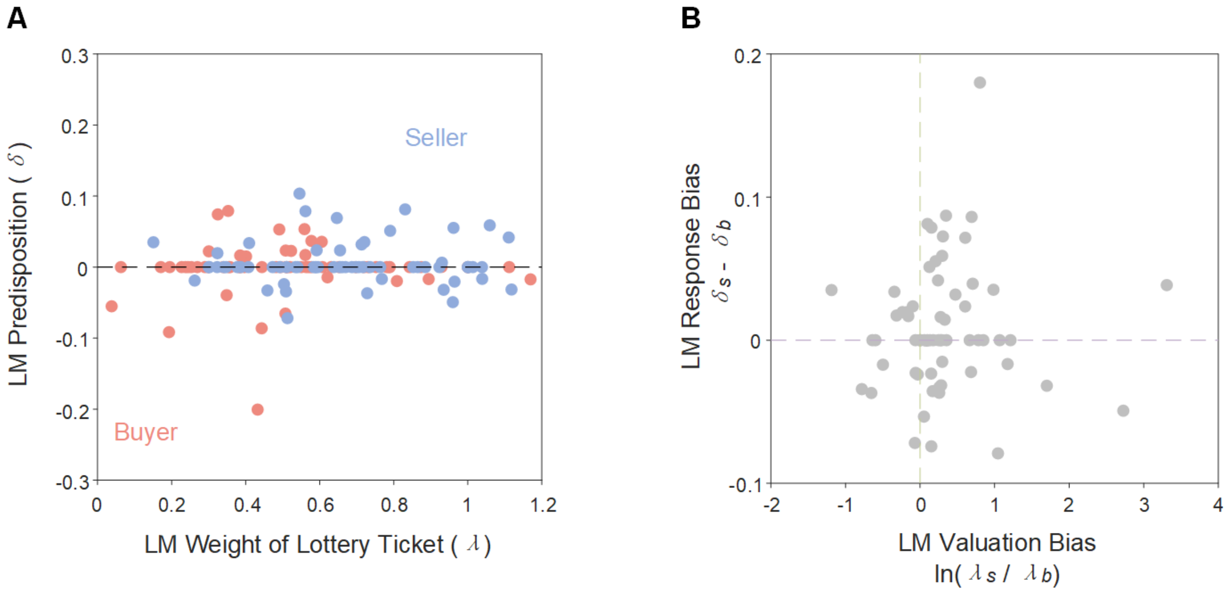

fig. S7. Parameter estimates of the logit model (LM1). (A) Estimated LM predisposition and LM lottery weights of buyers (red) and sellers (blue), respectively. (C) LM valuation bias and LM response bias. Each dot represents a participant.

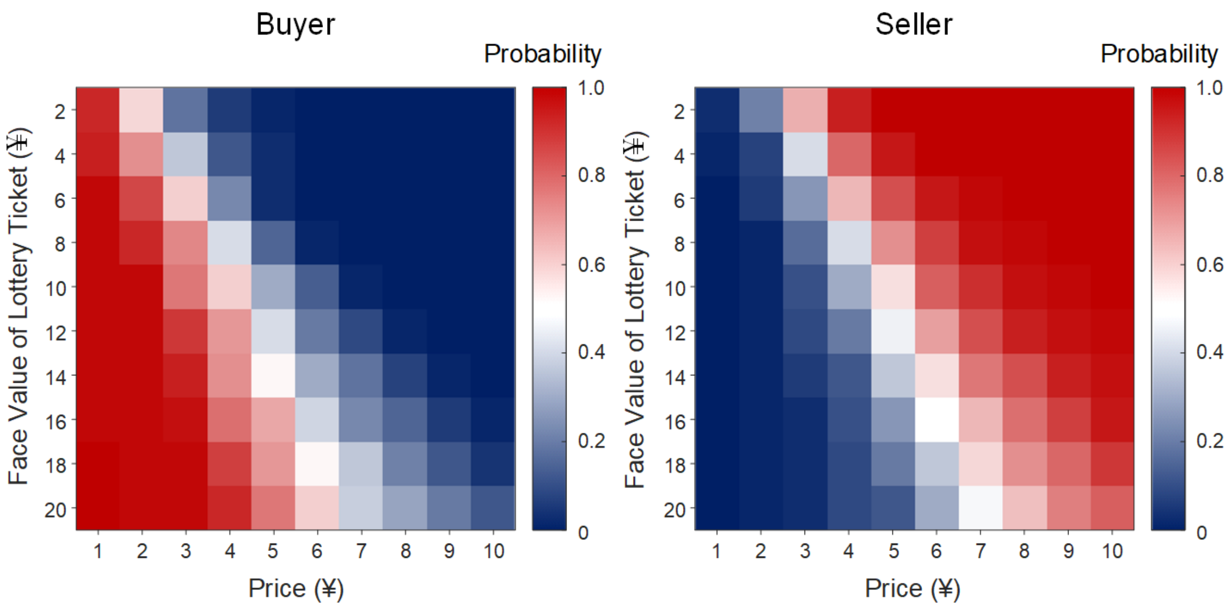

fig. S8. LM-predicted probability of acceptance across offers when participants acted as buyers (left) and sellers (right), respectively. Each cell denotes an offer. The x axes indicate prices, and the y axes indicate face values of lottery tickets.

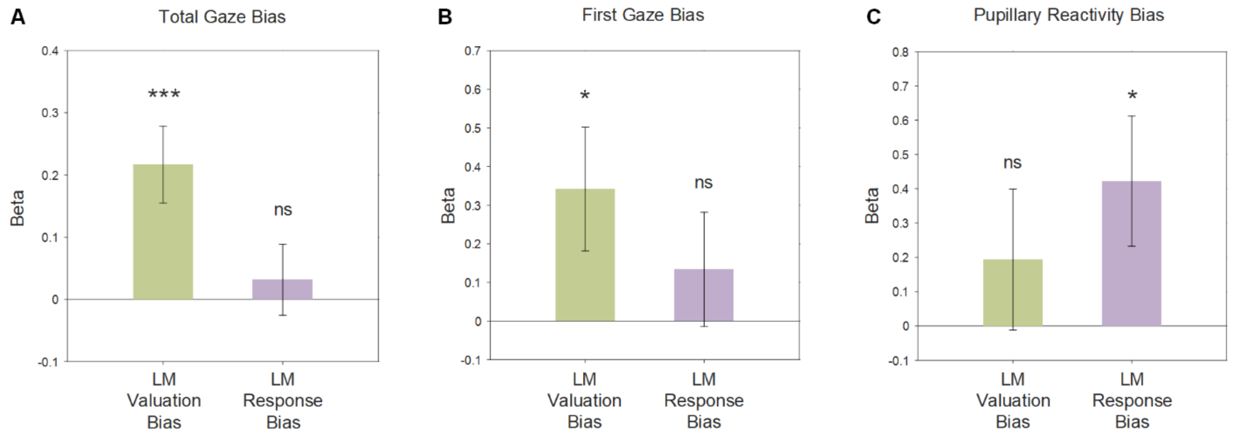

fig. S9. Estimates of regression models that used LM valuation bias and LM response bias to predict total gaze bias (A), first gaze bias (B) and pupillary reactivity bias (C), respectively. *: $p<0.05$; ***: $p<0.001$; ns: nonsignificant.

table S1. Summaries of mixed-level logistic regressions that used relative price to predict choices (accept=1, reject=0) of buyers (M1) and sellers (M2), respectively. Random effects of participants were included for relative price and constant. Standard errors of parameter estimates are displayed in parentheses. ***: p<0.001.

Model Index	M1 (Buyer)	M2 (Seller)
Relative Price	-0.893***	1.067***
	(0.041)	(0.051)
Constant	-0.857***	-0.698***
	(0.187)	(0.205)
Observations	6400	6400
R-squared	0.997	0.992
Adjusted R-squared	0.997	0.992

table S2. Summaries of DDMs. 95% credible intervals of parameter estimates are displayed in parentheses. DDM1 is the full model. DDM2, DDM3, DDM5 and DDM6 are the models that constrain the role effect on lottery weight, starting point, intercept or boundary separation, respectively. DDM4 is the model without intercept (i.e., *b*=0).

Model Index	Note	DIC	w_b	w_s	DDM Valuation Bias	z_b	z_s	DDM Response Bias	b_b	b_s	DDM Intercept Difference	a_b	a_s	Boundary Separation Difference	d	t
					$\ln(w_s/w_b)$			$z_s - z_b$			$b_s - b_b$			$a_s - a_b$		
DDM1	Full	26277	0.469	0.582	0.216	0.471	0.523	0.052	0.891	1.043	0.152	2.651	2.991	0.340	0.403	0.576
			(0.405, 0.537)	(0.513, 0.655)	(0.059, 0.376)	(0.456, 0.486)	(0.502, 0.545)	(0.033, 0.072)	(0.747, 1.042)	(0.864, 1.233)	(0.013, 0.294)	(2.474, 2.839)	(2.764, 3.225)	(0.195, 0.484)	(0.375, 0.431)	(0.532, 0.623)
DDM2	$w_b = w_s$	26423	0.530		0.000	0.470	0.522	0.052	0.753	1.156	0.403	2.657	2.957	0.300	0.401	0.577
			(0.469, 0.597)		(0.000, 0.000)	(0.454, 0.486)	(0.499, 0.544)	(0.032, 0.072)	(0.604, 0.903)	(0.953, 1.360)	(0.253, 0.547)	(2.481, 2.847)	(2.734, 3.189)	(0.155, 0.451)	(0.372, 0.429)	(0.534, 0.623)
DDM3	$z_b = z_s$	26426	0.468	0.578	0.211	0.499		0.000	0.816	1.114	0.298	2.643	2.984	0.340	0.401	0.569
			(0.403, 0.536)	(0.511, 0.652)	(0.055, 0.374)	(0.486, 0.511)		(0.000, 0.000)	(0.668, 0.964)	(0.929, 1.306)	(0.154, 0.445)	(2.464, 2.832)	(2.757, 3.217)	(0.193, 0.484)	(0.373, 0.431)	(0.525, 0.616)
DDM4	$b=0$	27731	0.748	0.930	0.217	0.502	0.564	0.062	0.000		0.000	2.542	2.905	0.364	0.316	0.582
			(0.666, 0.836)	(0.837, 1.023)	(0.090, 0.348)	(0.486, 0.517)	(0.542, 0.586)	(0.043, 0.081)	(0.000, 0.000)		(0.000, 0.000)	(2.368, 2.731)	(2.684, 3.137)	(0.223, 0.506)	(0.296, 0.336)	(0.537, 0.631)
DDM5	$b_b = b_s$	26369	0.447	0.605	0.303	0.467	0.527	0.060	0.962		0.000	2.643	2.991	0.348	0.400	0.576
			(0.384, 0.514)	(0.535, 0.680)	(0.150, 0.456)	(0.452, 0.482)	(0.506, 0.548)	(0.041, 0.079)	(0.825, 1.101)		(0.000, 0.000)	(2.462, 2.834)	(2.763, 3.225)	(0.201, 0.496)	(0.372, 0.429)	(0.533, 0.622)
DDM6	$a_b = a_s$	26859	0.490	0.561	0.136	0.461	0.518	0.057	0.855	1.091	0.236	2.828		0.000	0.399	0.565
			(0.421, 0.559)	(0.493, 0.631)	(-0.020, 0.294)	(0.444, 0.479)	(0.494, 0.543)	(0.036, 0.078)	(0.708, 1.007)	(0.903, 1.284)	(0.091, 0.388)	(2.645, 3.020)		(0.000, 0.000)	(0.370, 0.428)	(0.521, 0.611)

table S3. Summaries of linear regressions that used DDM valuation bias (M1), DDM response bias (M2), or both (M3) to predict total gaze bias. Order (buyer first=0, seller first=1) was controlled in M4. Each regressor was rescaled between 0 and 1. Standard errors are displayed in parentheses. *: p<0.05; **: p<0.01; ***: p<0.001.

Model Index	M1	M2	M3	M4
DDM Valuation Bias	0.164***		0.157***	0.159***
	(0.040)		(0.042)	(0.042)
DDM Response Bias		0.070	0.031	0.036
		(0.046)	(0.043)	(0.044)
Order				-0.009
				(0.019)
Constant	-0.055**	-0.016	-0.067*	-0.056
	(0.020)	(0.025)	(0.027)	(0.035)
Observations	64	64	64	64
R-squared	0.210	0.036	0.217	0.220
Adjusted R-squared	0.198	0.020	0.191	0.181

table S4. Summaries of aDDMs. 95% credible intervals of parameter estimates are displayed in parentheses. aDDM1 is the baseline aDDM. DDM1 is the full DDM without modeling gaze information. aDDM2 and aDDM3 are models allowing the modulatory effect of first gaze on boundary separation. aDDM4 and aDDM5 are models allowing the modulatory effect of first gaze on starting point. All models were estimated based on trials of valid gaze data. Consequently, the estimates of DDM1 here are not identical with the DDM1 estimates in **table S2**.

Model Index	Note	DIC	$w_{b,p}$	$w_{b,l}$	$w_{s,p}$	$w_{s,l}$	DDM Valuation Bias				z_b	z_s	DDM Response Bias	b_b	b_s	DDM Intercept Difference	a_b	a_s	Boundary Separation Difference	d	t	f_b	f_s	j_b	j_s
							Seller Gaze: Price		Seller Gaze: Lottery																
							Buyer Gaze: Lottery	Buyer Gaze: Price	Buyer Gaze: Lottery	Buyer Gaze: Price															
							$\ln(w_{s,p} / w_{b,l})$	$\ln(w_{s,p} / w_{b,p})$	$\ln(w_{s,l} / w_{b,l})$	$\ln(w_{s,l} / w_{b,p})$			$z_s - z_b$			$b_s - b_b$			$a_s - a_b$						
aDDM1	Baseline	25432	0.413	0.527	0.503	0.662	-0.048	0.200	0.228	0.476	0.469	0.522	0.053	0.904	1.055	0.151	2.641	2.996	0.355	0.403	0.585	0.000		0.000	
			(0.329, 0.500)	(0.455, 0.604)	(0.417, 0.597)	(0.583, 0.748)	(-0.257, 0.152)	(-0.052, 0.461)	(0.070, 0.393)	(0.266, 0.706)	(0.453, 0.484)	(0.501, 0.543)	(0.034, 0.073)	(0.751, 1.060)	(0.865, 1.245)	(0.012, 0.294)	(2.464, 2.830)	(2.769, 3.227)	(0.209, 0.501)	(0.374, 0.433)	(0.555, 0.617)	(0.000, 0.000)		(0.000, 0.000)	
DDM1	Non-aDDM	25479	0.467		0.583		0.223				0.469	0.525	0.055	0.903	1.047	0.144	2.635	2.984	0.349	0.404	0.585	0.000		0.000	
			(0.404, 0.535)		(0.515, 0.655)		(0.066, 0.382)				(0.454, 0.484)	(0.503, 0.545)	(0.036, 0.075)	(0.759, 1.052)	(0.867, 1.230)	(0.004, 0.286)	(2.460, 2.827)	(2.759, 3.218)	(0.203, 0.493)	(0.375, 0.433)	(0.555, 0.617)	(0.000, 0.000)		(0.000, 0.000)	
aDDM2	$f_b = f_s$	25380	0.415	0.527	0.505	0.661	-0.044	0.197	0.228	0.469	0.469	0.522	0.052	0.905	1.057	0.152	2.612	2.940	0.328	0.404	0.587	0.183		0.000	
			(0.334, 0.501)	(0.455, 0.603)	(0.418, 0.595)	(0.580, 0.746)	(-0.251, 0.158)	(-0.047, 0.451)	(0.069, 0.390)	(0.264, 0.691)	(0.454, 0.485)	(0.501, 0.543)	(0.033, 0.072)	(0.753, 1.063)	(0.869, 1.244)	(0.008, 0.298)	(2.440, 2.793)	(2.712, 3.167)	(0.182, 0.474)	(0.376, 0.433)	(0.557, 0.619)	(0.099, 0.271)		(0.000, 0.000)	
aDDM3	f_b, f_s	25389	0.415	0.526	0.503	0.662	-0.045	0.195	0.230	0.471	0.469	0.522	0.053	0.904	1.057	0.153	2.611	2.942	0.331	0.404	0.587	0.174	0.172	0.000	
			(0.332, 0.500)	(0.453, 0.600)	(0.420, 0.592)	(0.583, 0.747)	(-0.250, 0.159)	(-0.052, 0.449)	(0.074, 0.391)	(0.259, 0.697)	(0.454, 0.485)	(0.501, 0.544)	(0.033, 0.072)	(0.754, 1.055)	(0.872, 1.247)	(0.010, 0.298)	(2.441, 2.794)	(2.727, 3.170)	(0.185, 0.482)	(0.376, 0.433)	(0.556, 0.619)	(0.062, 0.294)	(0.079, 0.269)	(0.000, 0.000)	
aDDM4	$j_b = j_s$	25433	0.413	0.528	0.506	0.662	-0.045	0.203	0.227	0.475	0.468	0.520	0.052	0.904	1.054	0.150	2.644	2.997	0.353	0.403	0.585	0.000		0.005	
			(0.330, 0.498)	(0.456, 0.606)	(0.420, 0.597)	(0.585, 0.747)	(-0.259, 0.161)	(-0.046, 0.462)	(0.067, 0.386)	(0.266, 0.706)	(0.452, 0.483)	(0.499, 0.542)	(0.033, 0.072)	(0.751, 1.058)	(0.866, 1.245)	(0.007, 0.296)	(2.470, 2.834)	(2.780, 3.232)	(0.209, 0.501)	(0.374, 0.432)	(0.556, 0.617)	(0.000, 0.000)		(-0.008, 0.017)	
aDDM5	j_b, j_s	25432	0.416	0.525	0.503	0.661	-0.044	0.192	0.232	0.468	0.466	0.523	0.057	0.905	1.058	0.154	2.643	2.996	0.353	0.404	0.585	0.000		0.018	-0.004
			(0.331, 0.502)	(0.452, 0.601)	(0.416, 0.594)	(0.583, 0.747)	(-0.258, 0.163)	(-0.059, 0.454)	(0.074, 0.389)	(0.259, 0.694)	(0.450, 0.481)	(0.501, 0.545)	(0.038, 0.078)	(0.751, 1.061)	(0.869, 1.250)	(0.010, 0.300)	(2.465, 2.830)	(2.771, 3.230)	(0.207, 0.497)	(0.375, 0.433)	(0.555, 0.617)	(0.000, 0.000)		(-0.000,0.038)	(-0.020, 0.012)

table S5. Summaries of linear regressions that used DDM valuation bias (M1), DDM response bias (M2), or both (M3) to predict first gaze bias. Order (buyer first=0, seller first=1) was controlled in M4. Each regressor was rescaled between 0 and 1. Standard errors are displayed in parentheses. *: p<0.05.

Model Index	M1	M2	M3	M4
DDM Valuation Bias	0.265*		0.240*	0.230*
	(0.107)		(0.110)	(0.110)
DDM Response Bias		0.164	0.105	0.074
		(0.113)	(0.113)	(0.115)
Order				0.065
				(0.050)
Constant	0.029	0.065	-0.013	-0.025
	(0.054)	(0.063)	(0.071)	(0.071)
Observations	64	64	64	64
R-squared	0.090	0.033	0.103	0.127
Adjusted R-squared	0.076	0.017	0.074	0.084

table S6. Summaries of linear regression models that used first gaze bias (M1), total gaze bias (M2), or both (M3) to predict valuation bias. Order (buyer first=0, seller first=1) was controlled in M4. Each regressor was rescaled between 0 and 1. Standard errors are displayed in parentheses. *: $p<0.05$; **: $p<0.01$; ***: $p<0.001$.

Model Index	M1	M2	M3	M4
First Gaze Bias	0.590*		0.325	0.287
	(0.238)		(0.234)	(0.240)
Total Gaze Bias		1.066***	0.937**	0.948**
		(0.262)	(0.276)	(0.278)
Order				0.067
				(0.094)
Constant	0.022	-0.288*	-0.343*	-0.369*
	(0.100)	(0.137)	(0.142)	(0.147)
Observations	64	64	64	64
R-squared	0.090	0.210	0.235	0.241
Adjusted R-squared	0.076	0.198	0.210	0.203

table S7. Summaries of mixed-level regression models that used choice (1=accept, 0=reject), price, expected value of lottery ticket, or response time to predict decision-related pupil size. Random effects of participants were included for each regressor and constant. Each regressor was rescaled between 0 and 1. Standard errors are displayed in parentheses. *: p<0.05; ***: p<0.001.

Model Index	M1 (Buyer)	M2 (Buyer)	M3 (Buyer)	M4 (Buyer)	M5 (Buyer)	M6 (Buyer)	M7 (Seller)	M8 (Seller)	M9 (Seller)	M10 (Seller)	M11 (Seller)	M12 (Seller)
Choice	0.129***				0.171***	0.137***	0.058*				0.080*	0.079*
	(0.026)				(0.033)	(0.033)	(0.023)				(0.036)	(0.035)
Price		-0.085*			0.089	0.086		0.051			-0.031	-0.052
		(0.041)			(0.051)	(0.048)		(0.034)			(0.052)	(0.049)
Expected value of Lottery			0.072*		-0.003	-0.009			-0.010		0.036	0.019
			(0.031)		(0.034)	(0.034)			(0.030)		(0.034)	(0.034)
Reponse Time				6.440***		5.938***				3.831***		3.746***
				(0.920)		(0.855)				(0.716)		(0.710)
Constant	-0.382***	-0.292***	-0.370***	-0.511***	-0.444***	-0.591***	-0.320***	-0.321***	-0.290***	-0.469***	-0.334***	-0.484***
	(0.040)	(0.045)	(0.038)	(0.042)	(0.048)	(0.048)	(0.042)	(0.044)	(0.039)	(0.054)	(0.044)	(0.057)
Observations	6282	6282	6282	6282	6282	6282	6292	6292	6292	6292	6292	6292
R-squared	0.148	0.146	0.141	0.176	0.153	0.184	0.141	0.140	0.138	0.167	0.144	0.172
Adjusted R-squared	0.148	0.146	0.141	0.176	0.152	0.184	0.141	0.140	0.138	0.167	0.144	0.171

table S8. Summaries of linear regression models that used valuation bias (M1), response bias (M2), or both (M3) to predict pupillary reactivity bias. Order (buyer first=0, seller first=1) was controlled in M4. Each regressor was rescaled between 0 and 1. Standard errors are displayed in parentheses. *: $p<0.05$.

Model Index	M1	M2	M3	M4
DDM Valuation Bias	0.265		0.191	0.195
	(0.140)		(0.140)	(0.141)
DDM Response Bias		0.366*	0.319*	0.331*
		(0.141)	(0.144)	(0.148)
Order				-0.024
				(0.065)
Constant	0.083	0.017	-0.046	-0.041
	(0.071)	(0.078)	(0.090)	(0.091)
Observations	64	64	64	64
R-squared	0.055	0.098	0.125	0.127
Adjusted R-squared	0.039	0.084	0.096	0.083

table S9. Summaries of LMs. Standard deviations of parameter estimates are displayed in parentheses. LM1 is the full model. LM2, LM3 and LM5 are the models that constrain the role effect on lottery weight, predisposition or intercept, respectively. LM4 is the model without intercept (i.e., γ=0).

Model Index	Note	BIC	λ_b	λ_s	LM Valuation Bias<br>$\ln(\lambda_s / \lambda_b)$	δ_b	δ_s	LM Response Bias<br>$\delta_s - \delta_b$	γ_b	γ_s	LM Intercept Difference<br>$\gamma_s - \gamma_s$	μ
LM1	Full	6250	0.525	0.671	0.311	-0.002	0.006	0.008	-5.176	-6.522	-1.346	3.259
			(0.249)	(0.242)	(0.704)	(0.037)	(0.030)	(0.044)	(13.006)	(3.777)	(12.598)	(3.150)
LM2	$\lambda_b=\lambda_s$	6364	0.591		0.000	-0.007	0.007	0.015	-3.260	-6.743	-3.483	2.808
			(0.193)		(0.000)	(0.047)	(0.031)	(0.052)	(2.838)	(4.274)	(4.457)	(3.084)
LM3	$\delta_b=\delta_s$	6120	0.530	2.816	0.395	-0.003		0.000	-7.195	-5.992	1.203	4.248
			(0.255)	(17.117)	(0.974)	(0.042)		(0.000)	(16.406)	(3.627)	(15.761)	(10.594)
LM4	γ=0	7489	0.756	0.997	0.301	0.011	0.030	0.019	0.000		0.000	1.487
			(0.256)	(0.276)	(0.342)	(0.045)	(0.059)	(0.077)	(0.000)		(0.000)	(0.450)
LM5	$\gamma_b=\gamma_s$	6179	0.490	0.711	0.470	-0.006	0.013	0.018	-5.156		0.000	2.731
			(0.241)	(0.250)	(0.595)	(0.049)	(0.036)	(0.057)	(2.652)		(0.000)	(1.350)

table S10. Summaries of linear regressions that used LM valuation bias (M1), LM response bias (M2), or both (M3) to predict total gaze bias. Order (buyer first=0, seller first=1) was controlled in M4. Each regressor was rescaled between 0 and 1. Standard errors are displayed in parentheses. *: p<0.05; ***: p<0.001.

Model Index	M1	M2	M3	M4
LM Valuation Bias	0.218***		0.217***	0.217***
	(0.062)		(0.062)	(0.062)
LM Response Bias		0.038	0.031	0.032
		(0.062)	(0.057)	(0.058)
Order				0.003
				(0.019)
Constant	-0.053*	0.007	-0.063*	-0.068
	(0.023)	(0.023)	(0.029)	0.042
Observations	64	64	64	64
R-squared	0.168	0.006	0.172	0.173
Adjusted R-squared	0.155	-0.010	0.145	0.131

table S11. Summaries of linear regressions that used LM valuation bias (M1), LM response bias (M2), or both (M3) to predict first gaze bias. Order (buyer first=0, seller first=1) was controlled in M4. Each regressor was rescaled between 0 and 1. Standard errors are displayed in parentheses. *: p<0.05.

Model Index	M1	M2	M3	M4
LM Valuation Bias	0.347*		0.342*	0.338*
	(0.160)		(0.160)	(0.157)
LM Response Bias		0.145	0.134	0.147
		(0.152)	(0.148)	(0.146)
Order				0.087
				(0.049)
Constant	0.033	0.099	-0.011	-0.143
	(0.059)	(0.057)	(0.076)	(0.106)
Observations	64	64	64	64
R-squared	0.071	0.014	0.083	0.128
Adjusted R-squared	0.056	-0.001	0.053	0.085

table S12. Summaries of linear regressions that used LM valuation bias (M1), LM response bias (M2), or both (M3) to predict pupillary reactivity bias. Order (buyer first=0, seller first=1) was controlled in M4. Each regressor was rescaled between 0 and 1. Standard errors are displayed in parentheses. *: p<0.05.

Model Index	M1	M2	M3	M4
LM Valuation Bias	0.209		0.194	0.192
	(0.212)		(0.206)	(0.207)
LM Response Bias		0.429*	0.423*	0.427*
		(0.190)	(0.190)	(0.192)
Order				0.028
				(0.064)
Constant	0.132	0.058	-0.005	-0.047
	(0.078)	(0.071)	(0.098)	(0.139)
Observations	64	64	64	64
R-squared	0.015	0.076	0.089	0.092
Adjusted R-squared	0.000	0.061	0.059	0.047

table S13. Summaries of linear regressions that used LM response bias (M1), DDM response bias (M2), or both (M3) to predict pupillary reactivity bias. Order (buyer first=0, seller first=1) was controlled in M4. Each regressor was rescaled between 0 and 1. Standard errors are displayed in parentheses. *: p<0.05.

Model Index	M1	M2	M3	M4
LM Response Bias	0.429*		0.377*	0.375*
	(0.190)		(0.184)	(0.186)
DDM Response Bias		0.366*	0.333*	0.337*
		(0.141)	(0.138)	(0.144)
Order				-0.008
				(0.064)
Constant	0.058	0.017	-0.093	-0.083
	(0.071)	(0.078)	(0.093)	(0.125)
Observations	64	64	64	64
R-squared	0.076	0.098	0.156	0.156
Adjusted R-squared	0.061	0.084	0.129	0.114

table S14. Probability of choosing lottery ticket in each group and each block. Standard deviations are displayed in parentheses. 95% confidence intervals are displayed in square brackets. **: p<0.01; ***: p<0.001.

Order	Group 1 (Buyer First)		Group 2 (Seller First)		Between-Subject Difference (Seller-Buyer)
Block 1	Buyer:	0.395	Seller:	0.545	0.149***
		(0.134)		(0.136)	[0.937, 2.730]
Block 2	Seller:	0.521	Buyer:	0.409	0.112**
		(0.108)		(0.149)	[0.643, 2.349]
Within-Subject Difference (Seller-Buyer)		0.126***		0.136***	
		[0.074, 0.178]		[0.087, 0.185]	

table S15. Differential response time of choosing lottery ticket vs. choosing money in each group and each block. Standard deviations are displayed in parentheses. 95% confidence intervals are displayed in square brackets. *: p<0.05; **: p<0.01; ***: p<0.001.

Order	Group 1 (Buyer First)		Group 2 (Seller First)		Between-Subject Difference (Seller-Buyer)
Block 1	Buyer:	0.142 (0.391)	Seller:	-0.127 (0.376)	-0.269** [-0.460, -0.077]
Block 2	Seller:	-0.074 (0.397)	Buyer:	0.265 (0.456)	-0.339** [-0.553, -0.126]
Within-Subject Difference (Seller-Buyer)		-0.216* [-0.395, -0.038]		-0.392*** [-0.602, -0.181]	

table S16. Lottery weight in each group and each block. Standard deviations are displayed in parentheses. 95% confidence intervals are displayed in square brackets. *: p<0.05; **: p<0.01; ***: p<0.001.

Order	Group 1 (Buyer First)		Group 2 (Seller First)		Between-Subject Difference (Seller-Buyer)
Block 1	Buyer:	0.487<br>(0.173)	Seller:	0.617<br>(0.197)	0.130**<br>[0.037, 0.223]
Block 2	Seller:	0.576<br>(0.182)	Buyer:	0.472<br>(0.182)	0.103*<br>[0.012, 0.194]
Within-Subject Difference (Seller-Buyer)		0.088*<br>[0.021, 0.156]		0.145***<br>[0.070, 0.220]	

table S17. Starting point in each group and each block. Standard deviations are displayed in parentheses. 95% confidence intervals are displayed in square brackets. **: p<0.01; ***: p<0.001.

Order	Group 1 (Buyer First)		Group 2 (Seller First)		Between-Subject Difference (Seller-Buyer)
Block 1	Buyer:	0.475 (0.033)	Seller:	0.531 (0.050)	0.057*** [0.035, 0.078]
Block 2	Seller:	0.516 (0.055)	Buyer:	0.468 (0.059)	0.048** [0.019, 0.076]
Within-Subject Difference (Seller-Buyer)		0.041*** [0.024, 0.058]		0.063*** [0.046, 0.081]	

table S18. Intercept in each group and each block. Standard deviations are displayed in parentheses. 95% confidence intervals are displayed in square brackets. **: p<0.01.

Order	Group 1 (Buyer First)		Group 2 (Seller First)		Between-Subject Difference (Seller-Buyer)
Block 1	Buyer:	0.833	Seller:	1.068	0.236
		(0.460)		(0.562)	[-0.021, 0.492]
Block 2	Seller:	1.019	Buyer:	0.948	0.071
		(0.542)		(0.468)	[-0.182, 0.324]
Within-Subject Difference (Seller-Buyer)		0.186**		0.120	
		[0.054, 0.319]		[-0.006, 0.246]	

table S19. Boundary separation in each group and each block. Standard deviations are displayed in parentheses. 95% confidence intervals are displayed in square brackets. **: p<0.01; ***: p<0.001.

Order	Group 1 (Buyer First)	Group 2 (Seller First)	Between-Subject Difference (Seller-Buyer)
Block 1	Buyer: 2.723 (0.682)	Seller: 2.838 (0.851)	0.115 [-0.270, 0.500]
Block 2	Seller: 3.151 (0.879)	Buyer: 2.585 (0.815)	0.567** [0.143, 0.990]
Within-Subject Difference (Seller-Buyer)	0.428*** [0.215, 0.641]	0.254** [0.102, 0.405]	

table S20. Gaze-lottery ratio in each group and each block. Standard deviations are displayed in parentheses. 95% confidence intervals are displayed in square brackets.

Order	Group 1 (Buyer-Seller)		Group 2 (Seller-Buyer)		Between-Subject Difference (Seller-Buyer)
Block 1	Buyer:	0.500<br>(0.137)	Seller:	0.518<br>(0.111)	0.018<br>[-0.044, 0.081]
Block 2	Seller:	0.518<br>(0.120)	Buyer:	0.497<br>(0.102)	0.021<br>[-0.035, 0.076]
Within-Subject Difference (Seller-Buyer)		0.018<br>[-0.018, 0.054]		0.021<br>[-0.002, 0.044]	

table S21. First-gaze-lottery probability in each group and each block. Standard deviations are displayed in parentheses. 95% confidence intervals are displayed in square brackets. **: p<0.01; ***: p<0.001.

Order	Group 1 (Buyer First)	Group 2 (Seller First)	Between-Subject Difference (Seller-Buyer)
Block 1	Buyer: 0.178 (0.171)	Seller: 0.409 (0.260)	0.231*** [0.121, 0.341]
Block 2	Seller: 0.283 (0.217)	Buyer: 0.218 (0.212)	0.066 [-0.041, 0.173]
Within-Subject Difference (Seller-Buyer)	0.105** [0.029, 0.182]	0.191*** [0.123, 0.259]	

table S22. Different decision-related pupil size between choosing lottery ticket and choosing money in each group and each block. Standard deviations are displayed in parentheses. 95% confidence intervals are displayed in square brackets. **: p<0.01; ***: p<0.001.

Order	Group 1 (Buyer-Seller)	Group 2 (Seller-Buyer)	Between-Subject Difference (Seller-Buyer)
Block 1	Buyer: 0.152 (0.213)	Seller: -0.072 (0.187)	-0.224*** [-0.324, -0.124]
Block 2	Seller: -0.040 (0.200)	Buyer: 0.141 (0.250)	-0.180** [-0.293, -0.067]
Within-Subject Difference (Seller-Buyer)	-0.191*** [-0.292, -0.090]	-0.213*** [-0.303, -0.123]	
